# *Ameletus* Mayflies (Ephemeroptera: Ameletidae) of the Eastern Nearctic †

**DOI:** 10.3390/insects16050530

**Published:** 2025-05-16

**Authors:** David H. Funk

**Affiliations:** Stroud Water Research Center, Avondale, PA 19311, USA; dfunk@stroudcenter.org

**Keywords:** geographic parthenogenesis, polyploidy, hybridization, COI, allozymes, taxonomy

## Abstract

Fourteen *Ameletus* species are recognized in the eastern Nearctic (south of the Artic zone), including six described as new. Keys to adult males and full-grown larvae are provided. Taxonomic decisions were based on morphologic and genetic evidence. Their justification is discussed in depth and four species groups are proposed. The vast majority of *Ameletus* encountered in the eastern Nearctic are members of one of three triploid, clonal parthenogenetic species, at least two of which are of hybrid origin. Bisexual progenitors of the parthenogens were inferred using a combination of mitochondrial and nuclear genetic markers. The parthenogens likely arose during the Late Pleistocene when glacial advances brought previously allopatric species/populations into contact, and as glaciers retreated the parthenogens rapidly expanded their range while the sexual lineages remained in presumed glacial refugia. Although parthenogenesis is relatively common in Ephemeroptera, these *Ameletus* represent the first known cases of polyploidy and hybrid origin.

## Simple Summary

Aquatic insect species vary predictably in their ability to tolerate human-induced perturbations in freshwater ecosystems, and the presence or absence of species with known tolerance can be a strong indicator of water quality. The ability to accurately identify these species is therefore central to most water quality monitoring programs. *Ameletus* is a Holarctic genus of mayflies that typically inhabit cold mountain streams and whose presence is considered an indication of good to excellent water quality. As is true for many insects, accurate identification of *Ameletus* species relies primarily on structural differences in the adult male genitalia. However, in eastern North America, the vast majority of *Ameletus* specimens encountered are members of parthenogenetic species, which consist entirely of females whose eggs develop without the need for fertilization. The absence of males has hindered our ability to identify species from this region. This study uses morphology and genetics to resolve these parthenogens and their relationships to their sexually reproducing relatives and resulted in the discovery of six new species. Taxonomic keys are provided to enable the identification of both adults and larvae.

## 1. Introduction

*Ameletus* is a Holarctic genus with 61 currently recognized species worldwide. One species, *A. inopinatus* Eaton 1877, is found in northern Europe, Asia, and far northern North America [1]. Twenty-six species are exclusively Asian and 34 are North American. While there has been no comprehensive taxonomic treatment of the Palearctic fauna, Zloty [1] revised the Nearctic species based on adult males. In western North America, *Ameletus* are found from the extreme southwest U.S. north to Alaska, east to Nunavut, and in the East from Quebec to South Carolina and Alabama, but *Ameletus* are absent from a large area between these regions, from the Gulf of Mexico through the Great Plains. Because only one species (*A. subnotatus* Eaton 1885) is known in both the West and East, Zloty [1] presented two regional keys. His key to the Eastern species included only six of the eight known species, as males of the two most widespread and commonly collected species (*A. ludens* Needham 1905 and *lineatus* Traver 1932) were unknown. Since Zloty’s paper, a ninth Eastern species has been described, *A. janetae* Kondratieff and Meyer [2]. Herein, I describe an additional six new species and provide keys to larvae and adult males of the eastern Nearctic.

*Ameletus* larvae are cold stenotherms and are generally absent from waters whose temperature rises above about 15 °C. Thus, species found in southern regions are mostly restricted to cool, mountain streams and headwaters. However, three species (one of which is described as new herein), which have the broadest geographic ranges and together account for the vast majority of collections in eastern North America, have successfully colonized streams that become considerably warmer than 15° during the summer months. This expansion has been facilitated, at least in part, by the existence of an obligate summer egg diapause, enabling larvae to avoid exposure to warm summer temperatures. Together, these three species are also unique among *Ameletus* (as well as all other known ephemeropterans) in consisting entirely of polyploid, parthenogenetic clones. Their origin and biogeography are a major focus of this study.

The results are presented in the following order. Section 3.1 is a review of morphological characters used. Section 3.2 and Section 3.3 are taxonomic keys to male imagos and larvae. Section 3.4 is a formal taxonomic treatment of *Ameletus* in the eastern Nearctic. I recognize four species groups in this region, at least two of which are endemic. Species treatments are organized alphabetically by species group. Section 4.1 presents the evidence used for taxonomic decisions, especially regarding the *ludens* group, including evidence for the origins of the parthenogens. Section 4.2 includes a discussion of parthenogenesis in Ephemeroptera and its evolution in *Ameletus*. Section 4.3 is a discussion of the geography of parthenogenesis in *Ameletus*. The research behind this paper included detailed life history investigations, concentrating on voltinism and the roles of diapause and resistance to desiccation in the egg stage. Some of those results are included here, but details, especially regarding the egg stage, will be presented in a forthcoming paper.

## 2. Materials and Methods

### 2.1. Specimen Depositories: Abbreviations

The material examined has been deposited in the following institutions:

ANSP: Academy of Natural Sciences of Drexel University, Philadelphia, PA, USA

NHM: Natural History Museum, London, UK

CNC: Canadian National Collection, Ottawa, ON, Canada

CSUC: Colorado State University Collection, Fort Collins, CO, USA

CUIC: Cornell University Insect Collection, Ithaca, NY, USA

FAMU: Florida A&M University, Tallahassee, FL, USA

MU: Marshall University, Huntington, WV, USA

PERC: Purdue Entomological Research Collection, West Lafayette, IN, USA

SWRC: Stroud Water Research Center, Avondale, PA, USA

USNM: U.S. National Museum of Natural History, Washington, DC, USA

### 2.2. Laboratory Rearing and Culturing

Field-collected larvae were reared to the imago at the Stroud Water Research Center (SWRC), Avondale, Pennsylvania, USA, using techniques described in Sweeney et al. [3]. For specimens destined for genetic analysis, associated larval exuviae, imaginal legs (for DNA extraction), and abdomens were preserved in 95% ethanol and stored at 4 °C. Thoraces (for allozymes) and heads (for flow cytometry) were stored in cryovials at −80 °C. Except for types, all material is housed at the SWRC. Culturing of bisexuals was accomplished by inducing copulation (Figure 4G) using a technique similar to that described in Huff and McCafferty [4], and mated females were placed on water for oviposition (eggs dissected from the oviducts of mated females were found to have very low hatch rates compared to those from females allowed to oviposit naturally). Culturing of parthenogens generally involved the dissection of eggs from imagos, as these females rarely oviposited when placed on water. The eggs were incubated in sterile 30 mL museum jars containing 10 mL filtered (0.45 µm), sterilized, stream water taken from White Clay Creek (WCC) at SWRC. Jars were incubated at either 10 °C in the dark or at ambient WCC temperature and photoperiod.

### 2.3. Allozymes

Homogenates from imaginal thoraces were electrophoresed in horizontal starch gels as described in Sweeney et al. [5]. Table S1 lists enzymes and running conditions used for *Ameletus*. Data were analyzed using POPTREE2 [6]. For triploid parthenogenetic clones, if three alleles were detected in an individual, frequencies were each entered as 0.333; when two alleles were present with obvious dosage differences, they were scored as 0.333 and 0.667; when two alleles were present and dosage could not be determined, each was scored as 0.5. Expected heterozygosities for sexuals were calculated in POPTREE2; for parthenogens, heterozygosity was determined by direct count. Neighbor joining trees were constructed from genetic distances (D_A_; Nei et al. [7]). *n*-values for bisexual species were as follows: *ohioensis* (62), *janetae* (7), *burkei* (38), *tarteri*-Shay (29), *tarteri*-Ham (45), *matrilineatus* (37), and *patriludens* (25).

### 2.4. Flow Cytometry

Genome size was determined from the head tissue of *Ameletus* imagos using methods described in Hare and Johnston [8]. Flow cytometry was performed on a Beckman Coulter CytoFLEX LX Flow Cytometer, using heads of female *Drosophila melanogaster* and chicken red blood cells as sizing standards. Ploidy determination of parthenogens was based on genome size (pg DNA/nucleus) relative to that of bisexuals (which were presumed to be diploid).

### 2.5. Genetic Barcoding

Sequencing of mitochondrial cytochrome c oxidase subunit 1 (COI) was performed by the Canadian Centre for DNA Barcoding (CCDB) on 450 specimens collected during this study, and the data are managed in the Barcode of Life Database (BOLD), project DHF (SWRC-Mayflies). Public sequences from an additional 72 specimens from the eastern Nearctic, plus representatives from each of the other 82 Holarctic Ameletidae Barcode Index Numbers (BINs) available as of January 2025, were downloaded from BOLD and used for phylogenetic analysis. Analyses were performed with MEGA12 software [9] to produce maximum likelihood trees using the Adaptive Bootstrap test with a threshold of 5. Fasta files for the two most common parthenogenetic haplotypes are provided in Supplementary Materials.

### 2.6. Imaging

Most adult habitus photos were taken with a Nikon D90 digital SLR using a 55 mm Micro Nikkor lens and an SB-25 flash. Larval in-stream habitus photos were taken using an underwater rig (custom-designed and constructed by the author) employing a 105 mm Micro Nikkor lens and a modified Vivitar 285 flash. Photomicrographs of slide-mounted male genitalia were made with a Nikon Labophot-2 microscope with a trinocular head, using a Canon T1i digital SLR. Images were captured using Canon EOS Utility 2 software and stacked manually as needed using Affinity Photo 2 software.

## 3. Results

This paper covers the eastern Nearctic region south of the Arctic (i.e., east of longitude −95° and south of latitude 60°). *Ameletus inopinatus* Eaton 1887, a Holarctic species, has been found from Alaska across northern Canada to eastern Nunavut. Readers are referred to Zloty [1] and Zloty and Pritchard [10] for treatment of this species.

### 3.1. Morphological Characters Used in Keys and Descriptions

#### 3.1.1. Imagos

The identification of adult *Ameletus* species depends, to a large degree, on characters of the male genitalia. Thus, with the exception of *A. subnotatus*, which has distinctively speckled wings in both sexes, female imagos from the eastern Nearctic cannot generally be identified beyond genus and are not keyed here.

For male genital terminology, I mostly follow that of Zloty [1]. See Figure 12B–E for a labeled illustration of the important structures. Like other mayflies, *Ameletus* males have paired genital openings (gonopores) and the intromittent organs, which are fused to some degree at their base, are sometimes referred to in the singular (penis) and sometimes pleural (penes). I use the latter. Dorsal sclerotized extensions beyond the gonopores are referred to as the lateral lobes, and beneath are the ventral plates. The genital forceps (=gonostyli of Kluge [11]) are three segmented in the imago, but the basal segment appears subdivided and, to avoid confusion, is referred to herein as the long joint. For Figures B–D in most of the species plates, I photographed slide-mounted penes and styliger plates separately. The penes are shown in ventral view and the styliger plate in dorsal view. The taxonomically important features of the ventral plates of the penes include whether or not they are produced into a distal spine, and the presence and size of accessory teeth along their ventral edge. For species other than *A. subnotatus* and members of the *A. browni* group, the shape and size of the terminal spine are best visualized in lateral view (as in Figure 15A–G).

#### 3.1.2. Larvae

Full-grown larval body size is quite variable among *Ameletus* in the eastern Nearctic, but most of this variation is intraspecific. Like most univoltine mayflies, *Ameletus* individuals that mature early in the emergence season are much larger on average than those emerging later in the season. For example, for *A. ludens* and *A. lineatus* in White Clay Creek, Pennsylvania, adult dry mass at the beginning of the emergence period is as much as 8 times that seen at the end of the emergence period (see Figure 21) which corresponds to a decrease in full-grown larval body length from 14.5 mm down to 7.5 mm. That range is equivalent to the range of body sizes seen in all the eastern Nearctic species combined. Also, for a given species, body size also varies considerably among local populations. For these reasons, body size is of little value in distinguishing species and is not used in the key.

All eastern Nearctic *Ameletus* larvae have rows of spinules (or microspines) along the posterior margins of at least the last five abdominal tergites, but the presence or absence of these on more anterior tergites varies among species and can thus be useful in distinguishing them. However, their presence also varies developmentally; species characterized by spinules on more anterior segments lack them in earlier instars. Thus, this character is only useful for fully grown larvae.

*Ameletus* larvae have seven pairs of gills (=tergalii of Kluge [11]) on abdominal segments 1–7 that vary in size and shape depending on which segment they arise from. There are differences among species that are taxonomically useful. These are best observed on gill 4, but gills 3 or 5 will also suffice. In life, the gills project perpendicularly from the abdomen and the plane of the gill is oriented vertically, with the anal (posterior) margin facing upward (e.g., see Figure 5B). One sclerotized rib (or band) runs along the costal (anterior) margin and another along or near the anal (posterior) margin of the gill (see arrow in Figure 3G). The anal rib (which Zloty and Pritchard [10] refer to as the mesal band) is inset to varying degrees in some species (e.g., Figure 5E). The region between that rib and the anal edge is what Zloty and Pritchard [10] refer to as the mesal extension. A narrowly inset anal rib helps to define the *A. ludens* goup, but this feature is only evident in fully grown larvae. In most species there is a row of robust spines (in addition to the fine setae) along both the costal and anal margins of the gill, and the absence of spines on the anal margin characterizes *A. tertius*.

On the larval mandible, there appears a row of long, fine setae along the mesal edge, between the incisor (and prostheca) and the molar area (see Figure 12F). In most species, there is a gap between the proximal end of the setal row and the brush of setae in the molar region (the molar brush). The presence and width of this gap (relative to the length of the setal row) vary among species groups. In the key (and figures), I refer to measurements made on the left mandible. The mandibles are not bilaterally symmetrical, but the right mandible shows a similar setal pattern. The mandible must usually be removed to adequately see this character, but I use it early in the key because it is useful for distinguishing species groups and, unlike the gill and tergal spine characters above, can be used for smaller (down to about half-grown) larvae.

Color patterns, especially those on the dorsum of the abdomen, vary considerably among individuals as well as developmentally. But, some patterns, such as the presence of ventral stripes on the abdomen or a regular alternation of dark- and light-colored segments in the caudal filaments (“speckling”), are important characters for distinguishing species. However, the ventral stripes in particular vary developmentally and thus are only useful for larvae that have reached the final or penultimate instar (see Figure 16A–D).

Morphologically, the three species of obligate parthenogens can only be distinguished from some of their bisexual relatives by the absence of males. Thus, it is important to determine the gender of larvae. This can be determined by the shape of the distal margin of the ninth sternite (see Figure 17A–E). In male larvae, precursors to the adult styliger plate and genital forceps become visible by the time larvae are about half grown (in terms of body length).

### 3.2. Key to Male Imagos


1.Penes with lateral lobes short and broad, not reaching to the base of the forceps (Figure 13D).............................................................*subnotatus* group 2

—Penes with lateral lobes longer, reaching beyond base of forceps (similar to Figure 13E)....................................................................................................3

2.Forewings clear with dark brown to black blotches, giving them a speckled appearance (Figure 13A); spine-like lateral lobe of penes with a conspicuous large tooth near base (Figure 13B)...........................................................................................................................................................*subnotatus*

—Forewings without dark blotches; spine-like lateral lobes without tooth near base (Figures 35B and 36B in Zloty [1]).........................................*walleyi*

**Figure 1:**
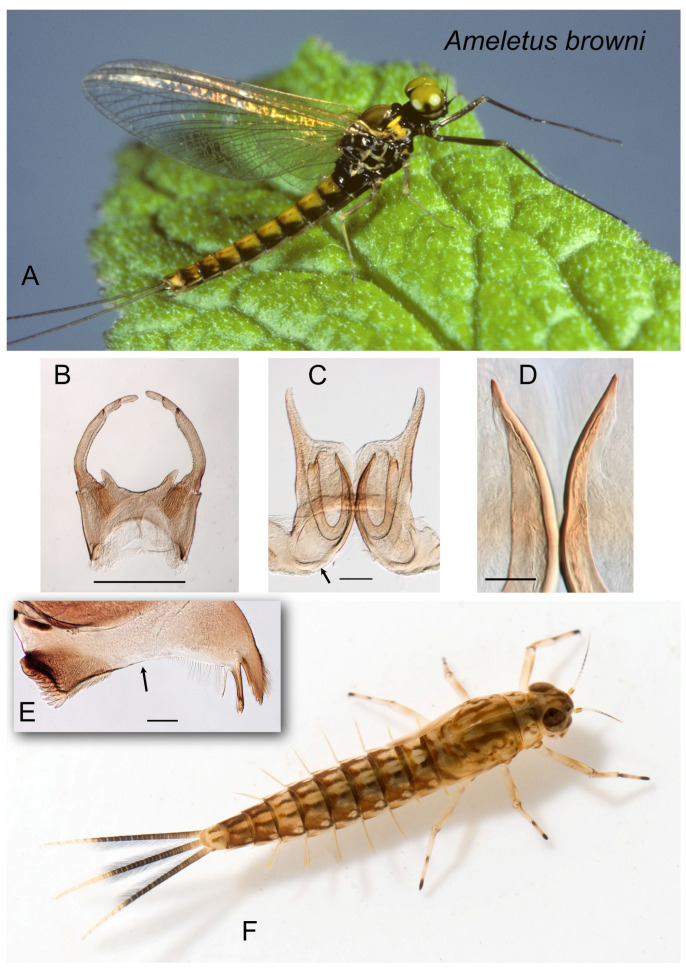
*Ameletus browni*. (**A**). Living male imago, lateral view. (**B**). Male styliger plate and forceps, dorsal view. Scale = 0.5 mm. (**C**). Penes of male, ventral view. Arrow indicates basal sclerite. Scale = 0.1 mm. (**D**). Ventral plates of penes, ventral view. Scale = 0.05 mm. (**E**). Left mandible of larva, dorsal view. Arrow indicates setal gap. Scale = 0.1 mm. (**F**). Living larva, dorsal view.

**Figure 2:**
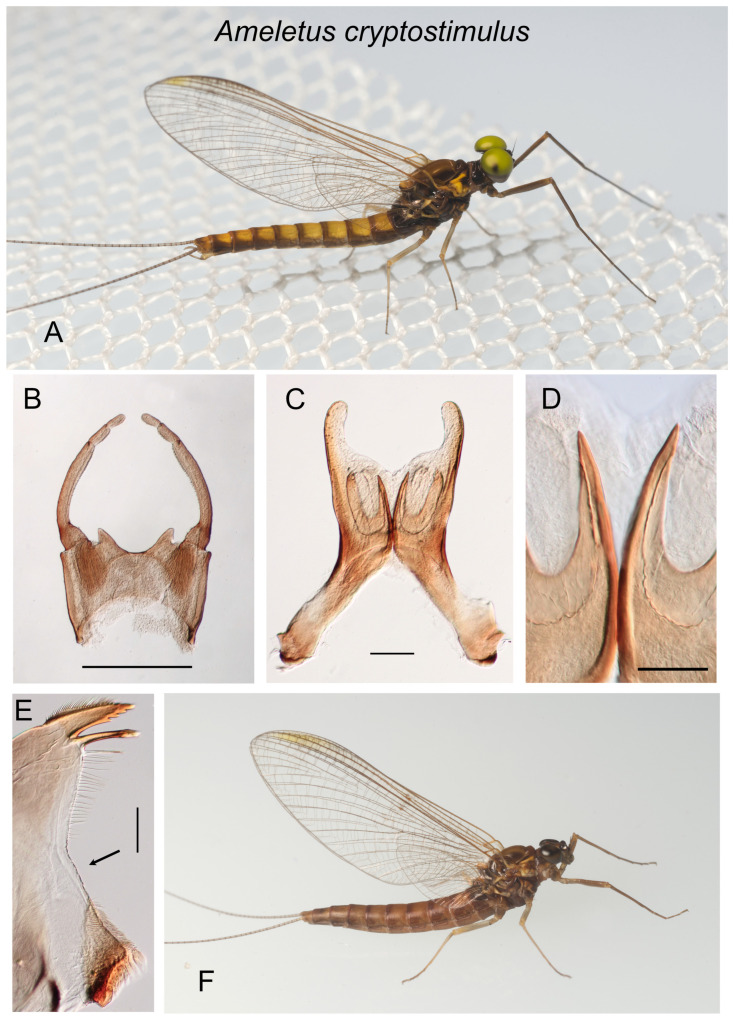
*Ameletus cryptostimulus*. (**A**). Living male imago, lateral view. (**B**). Male styliger plate and forceps, dorsal view. Scale = 0.5 mm. (**C**). Penes of male, ventral view. Scale = 0.1 mm. (**D**). Ventral plates of penes, ventral view. Scale = 0.05 mm. (**E**). Left mandible of larva, dorsal view. Arrow indicates setal gap. Scale = 0.1 mm. (**F**) Living female imago, lateral view.

**Figure 3:**
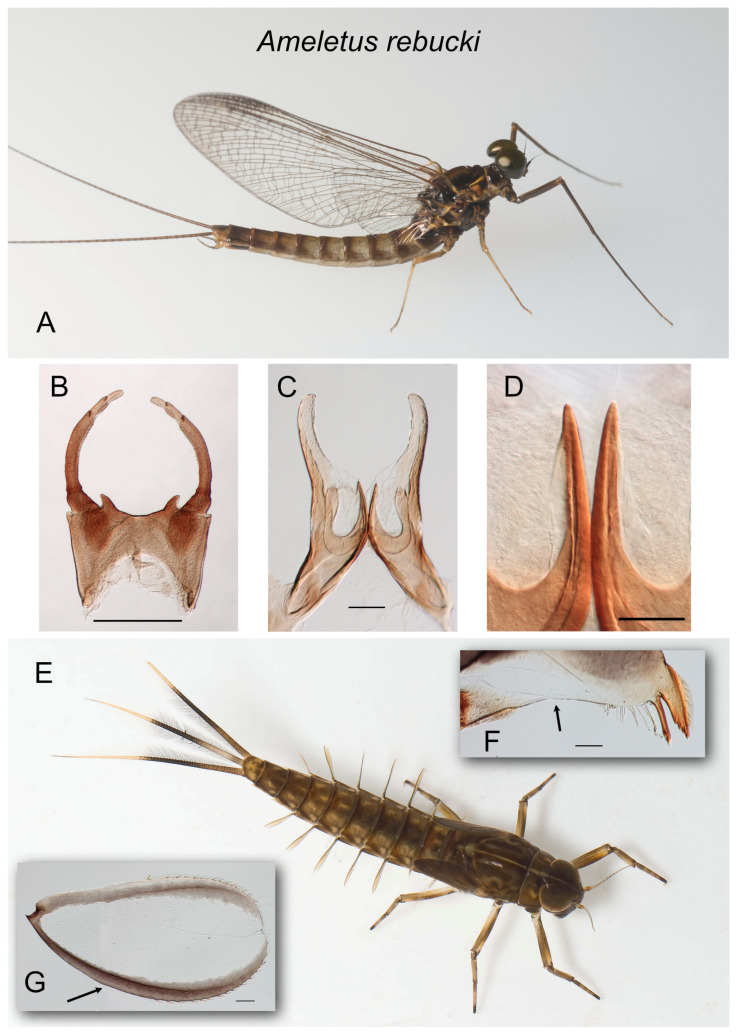
*Ameletus rebucki*. (**A**). Living male imago, lateral view. (**B**). Male styliger plate and forceps, dorsal view. Scale = 0.5 mm. (**C**). Penes of male, ventral view. Scale = 0.1 mm. (**D**). Ventral plates of penes, ventral view. Scale = 0.05 mm. (**E**). Living larva, dorsal view. (**F**). Left mandible of larva, dorsal view. Arrow indicates setal gap. Scale = 0.1 mm. (**G**). Larval gill 4 (right). Arrow indicates anal rib, which is not inset. Scale = 0.1 mm.

3.Ventral plates of penes forming a distinctive U-shaped structure, with an acute median terminal spine and no accessory teeth (panels C and D in Figure 1, Figure 2 and Figure 3).............................................................................................................................................................................*browni* group 4

**Figure 4:**
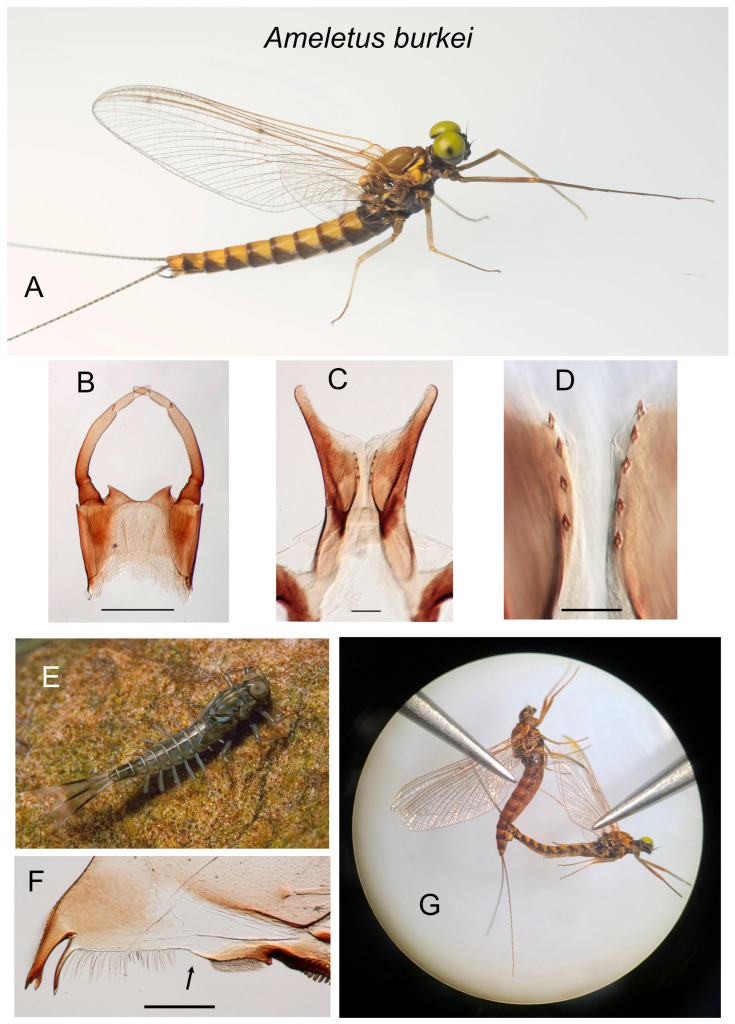
*Ameletus burkei*. (**A**). Living male imago, lateral view. (**B**). Male styliger plate and forceps, dorsal view. Scale = 0.5 mm. (**C**). Penes of male, ventral view. Scale = 0.1 mm. (**D**). Ventral plates of penes, ventral view. Scale = 0.05 mm. (**E**). Living larva, dorsolateral view. (**F**). Right mandible of larva, dorsal view. Arrow indicates setal gap. Scale = 0.1 mm. (**G**). Female (above) and male imagos during laboratory-induced copulation.

—Ventral plates of penes not forming such a U-shaped structure, and with at least a few accessory teeth along its ventral margin (as in panels C and D in Figure 4 and Figure 11).........................................................................................................................................................................................................6

4.Penes with narrow lateral lobes that are shorter relative to the U-shaped ventral plate; base of penes broad and produced antero-medially (Figure 1C)............................................................................................................................................................................................................................................*browni*

—Penes with wider lateral lobes that are longer relative to the U-shaped ventral plate; base of penes narrow and not produced anteriorly (Figure 2C and Figure 3C)................................................................................................................................................................................................................................5

5.Lateral lobes of penes distinctly longer than the lateral arm of the U-shaped ventral plate (Figure 3C); tergites predominantly light gray-brown with darker brown posterior and lateral margins (Figure 3A); upper portion of compound eye dark brown with only a hint of greenish coloration in life (Figure 3A); range northern New England south to West Virginia......................................................................................................................*rebucki*

—Lateral lobes of penes not or only slightly longer than the lateral arm of the U-shaped ventral plate (Figure 2C); tergites predominantly light yellow-brown with darker brown posterior and lateral margins (Figure 2A); upper portion of compound eye light yellowish green in life (Figure 2A); range Virginia to South Carolina ..................................................................................................................................................................*cryptostimulus*

6.Long joint of forceps with a prominent (as in Figure 9B) or subtle (Figure 6B) dilation in the apical 1/3rd........................................................................7

—Long joint of forceps without dilation in the apical 1/3rd (panel B in Figure 4, Figure 10, Figure 12 and Figure 15).......................................................9

7.Dilation in the long joint of forceps subtle (Figure 6B); ventral plates of penes produced distally into a very small spine (best viewed laterally, Figure 15D) ............................................................................................................................................................................................................................*janetae*

—Dilation in the long joint of forceps more pronounced (Figure 9B and Figure 11B); ventral plates of penes produced distally into a more prominent spine (Figure 15E or Figure 15F)..................................................................................................................................................................................................8

8.Terminal spines of ventral plates of penes shorter (Figure 15E); known from New York and Pennsylvania......................................................*patriludens*

—Terminal spines of ventral plates of penes longer (Figure 15F); known only from southwestern Virginia.....................................................*matrilineatus*

9.Ventral plate of penes produced into a prominent spine distally (best viewed laterally, Figure 15G).........................................................................*tarteri*

—Ventral plate of penes not produced into a spine distally (Figure 15A–C)...........................................................................................................................10

10.Ventral plate of penes with a basal lobe projecting anteriorly (Figure 14C); spinules on ventral margin of ventral plate of penes longer, about 20 µm in length (range 18–23 µm) (Figure 14D)....................................................................................................................................................................*tertius*

—Ventral plate of penes without a basal lobe projecting anteriorly (Figure 4C and Figure 10C); spinules on ventral margin of ventral plate of penes shorter, 10–12 µm in length (Figure 4D and Figure 10D)........................................................................................................................................................11

11.Ventral plate of penes wider in lateral view (Figure 15C); known only from southwestern Virginia........................................................................*burkei*

—Ventral plate of penes narrower in lateral view (Figure 15B); range southeastern Ohio and adjacent West Virginia...........................................*ohioensis*


### 3.3. Key to Full-Grown Larvae (Walleyi Unknown)


1.Mandible without a gap between the molar brush and the row of fine setae extending to the prostheca and incisor (i.e., no setal gap; Figure 14I); anal rib of gill 4 not inset from margin and with robust spines along only the costal margin (Figure 14H)..............................................................*tertius*

—Mandible with a distinct setal gap that is at least 0.2× the length of the setal row (as in Figure 7C); anal rib of gill 4 inset or not inset from anal margin, but with spines on both the costal and anal margins.................................................................................................................................................2

2.Setal gap on mandible wide, usually >0.8× the length of the setal row (as in Figure 13G); anal rib of gill 4 inset far from anal margin (Figure 13F) or not inset at all (as in Figure 3G)...............................................................................................................................................................................................3

**Figure 5:**
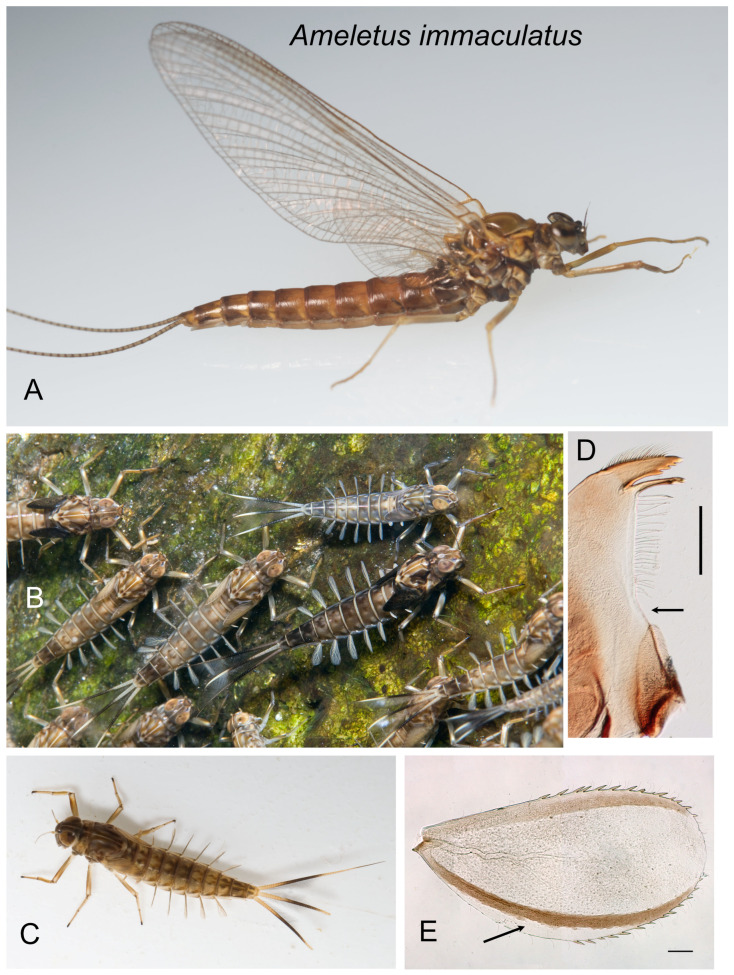
*Ameletus immaculatus*. (**A**). Living female imago, lateral view. (**B**). Group of living female larvae on the stream bottom. (**C**). Living female larva, dorsal view. (**D**). Left mandible of larva, dorsal view. Arrow indicates setal gap. Scale = 0.1 mm. (**E**). Larval gill 4 (right), dorsal view. Arrow indicates where anal rib is inset. Scale = 0.1 mm.

—Setal gap narrow, <0.5× length of the setal row (as in Figure 12F); anal rib of gill 4 narrowly inset on full-grown larvae (as in Figure 5E)............................................................................................................................................................................................................................*ludens* group 6

3.Caudal filaments with dark band usually extending from base to about the distal ¾, with every 4th segment pale giving them a speckled appearance (Figure 13H); anal rib of gill 4 inset far from anal margin (Figure 13F); northern transcontinental species...................................*subnotatus*

—Caudal filaments with dark band either extending from base, or restricted to middle 2/3, but with pigmentation uniform within the band (Figure 1F and Figure 3E); anal rib of gill 4 not inset from anal margin (Figure 3G); range New England south to South Carolina...................*browni* group 4

4.Spinules present on posterior margins of tergites 1–10; dorsum of abdomen usually with a distinct median pale stripe (Figure 1F); caudal filaments usually dark from base, with middle 2/3 only slightly darker; Quebec and Maritimes south to northern Pennsylvania.......................*browni*

—Spinules absent from posterior margins of tergites 1 and 2; dorsum of abdomen without a pale stripe; caudal filaments usually with a distinct dark band in middle 1/3 (basal 1/3 may be darkened somewhat; Figure 3E); New England south to South Carolina....................................................5

5.Spinules present on posterior margins of tergites 5– or 6–10 in late instar larvae; range Virginia, North Carolina, eastern Tennessee, and South Carolina.....................................................................................................................................................................................................................*cryptostimulus*

—Spinules present on posterior margins of tergites 3– or 4–10 in late instar larvae; range from Maine south to West Virginia..............................*rebucki*

**Figure 6:**
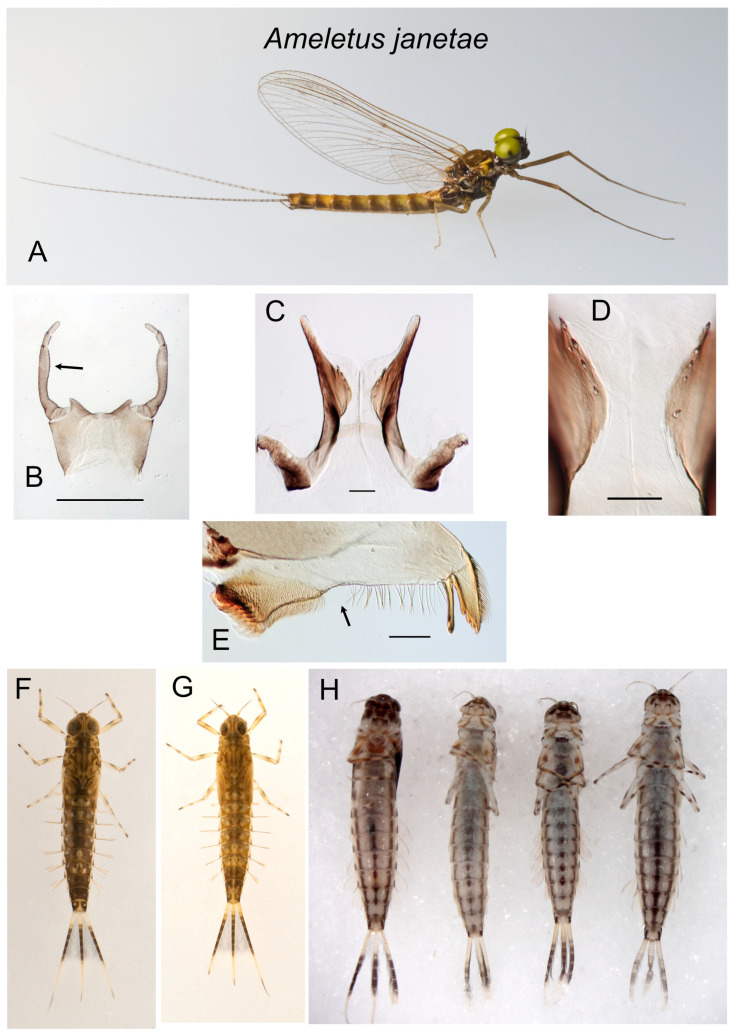
*Ameletus janetae*. (**A**). Living male imago, lateral view. (**B**). Male styliger plate and forceps, dorsal view. Arrow indicates dilation of long joint. Scale = 0.5 mm. (**C**). Penes of male, ventral view. Scale = 0.1 mm. (**D**). Ventral plates of penes, ventral view. Scale = 0.05 mm. (**E**). Left mandible of larva, dorsal view. Arrow indicates setal gap. Scale = 0.1 mm. (**F**). Living larva, dorsal view. (**G**). Living larva, dorsal view. (**H**). Larvae, ventral view, showing variation in ventral abdominal striping.

6.Caudal filaments with a distinct dark band in the middle 1/3, with every 4th segment paler, giving the band a speckled appearance (as in Figure 9F); full-grown larvae with 3 dark stripes on the venter of the abdomen (Figure 16B,C,E and Figure 6H).............................................*ludens* complex 7

—Caudal filaments with dark band uniformly pigmented; venter of abdomen may have dark maculae but usually without stripes (approaching stripes in some *ohioensis*; see Figure 10F).......................................................................................................................................................*tarteri* complex 11

7.Spinules present on posterior margins of tergites 4–10 in late instars (absent from 1–2, sometimes scattered minute spinules on 3); sternal stripes not coalescing on sternite 9 (Figure 6H); known distribution limited to a small area in eastern West Virginia........................................................*janetae*

—Spinules present on posterior margins of tergites 1–10 in late instars; sternal stripes coalescing on sternite 9 (Figure 16B,C,E); widely distributed, from Quebec and Newfoundland south to South Carolina and Georgia, west to Arkansas................................................................................................8

8.Sternal stripes in last instar larvae with margins distinct; width of median stripe subequal to or only slightly wider than lateral stripes; stripes not coalescing until distal 1/3 of sternite 9 (right side of Figure 16E).............................................................................................................................................9

—Sternal stripes in last instar larvae with margins less distinct (especially the median stripe); median stripe distinctly wider than lateral stripes; stripes coalescing midway on sternite 8 and in the basal 1/3 or sternite 9 (left side of Figure 16E)..................................................................................10

9.Bisexual, with males and females present in approximately equal proportion (see Figure 17A–E for determination of sex in larvae); known only from southwest Virginia............................................................................................................................................................................................*matrilineatus*

—Triploid parthenogen, with a complete absence of males; widespread from northern Pennsylvania south to South Carolina and Georgia, west to Arkansas..............................................................................................................................................................................................................................*lineatus*

10.Bisexual, with males and females present in approximately equal proportion (see Figure 17A–E for determination of sex in larvae); known only from southern New York and northern Pennsylvania.............................................................................................................................................*patriludens*

—Triploid parthenogen, with a complete absence of males; widespread from Quebec and Newfoundland south to West Virginia.......................*ludens*

11.Sternites with variable pattern of dark mottling (Figure 10E–F); bisexual, with males and females in approximately equal proportions; known from southeastern Ohio and adjacent West Virginia...................................................................................................................................................*ohioensis*

—Without distinct color pattern on sternites (although there may be some darkening, especially near lateral margins); bisexual or parthenogenetic; widespread from northern New England to South Carolina, west to at least West Virginia and Ohio............................................................................12

12.Triploid parthenogen, with males completely absent (see Figure 17A–E for determining sex of larvae).......................................................*immaculatus*

—Bisexual, with males and females in approximately equal proportion.................................................................................................................................13

13.Range eastern West Virginia and western Maryland; with minimal dark shading on sternites; often (but not exclusively) found in streams with very low pH (<5)..................................................................................................................................................................................................................*tarteri*

—Known only from southwest Virginia; some specimens with more extensive dark shading on sternites; probably intolerant of low pH............*burkei*


**Figure 7:**
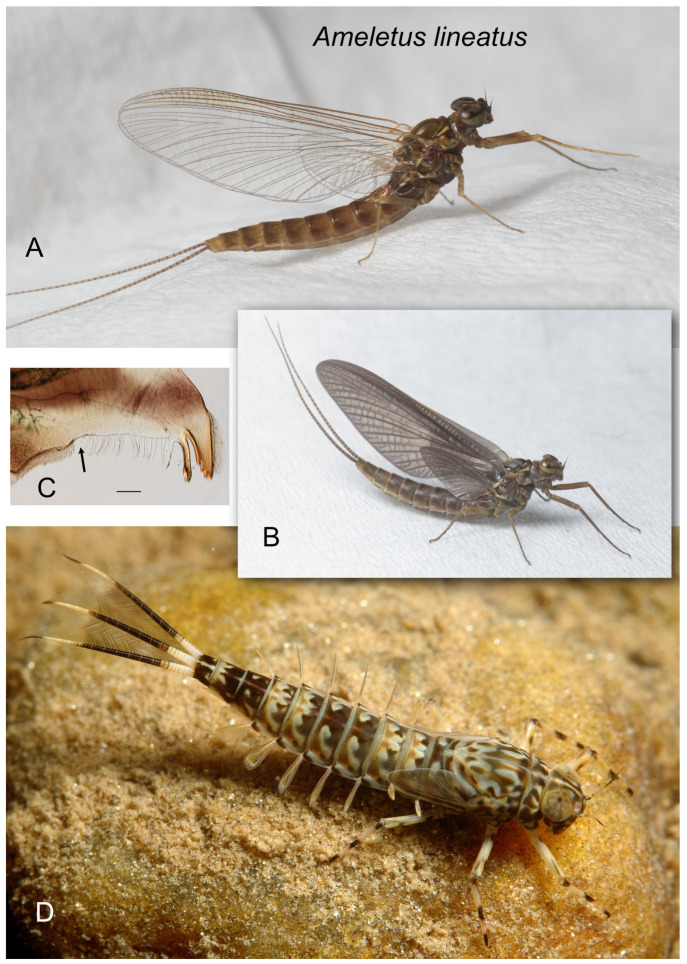
*Ameletus lineatus*. (**A**). Living female imago, lateral view. (**B**). Living female subimago, lateral view (**C**). Left mandible of larva, dorsal view. Arrow indicates setal gap. Scale = 0.1 mm. (**D**). Living female larva, dorsolateral view.

**Figure 8:**
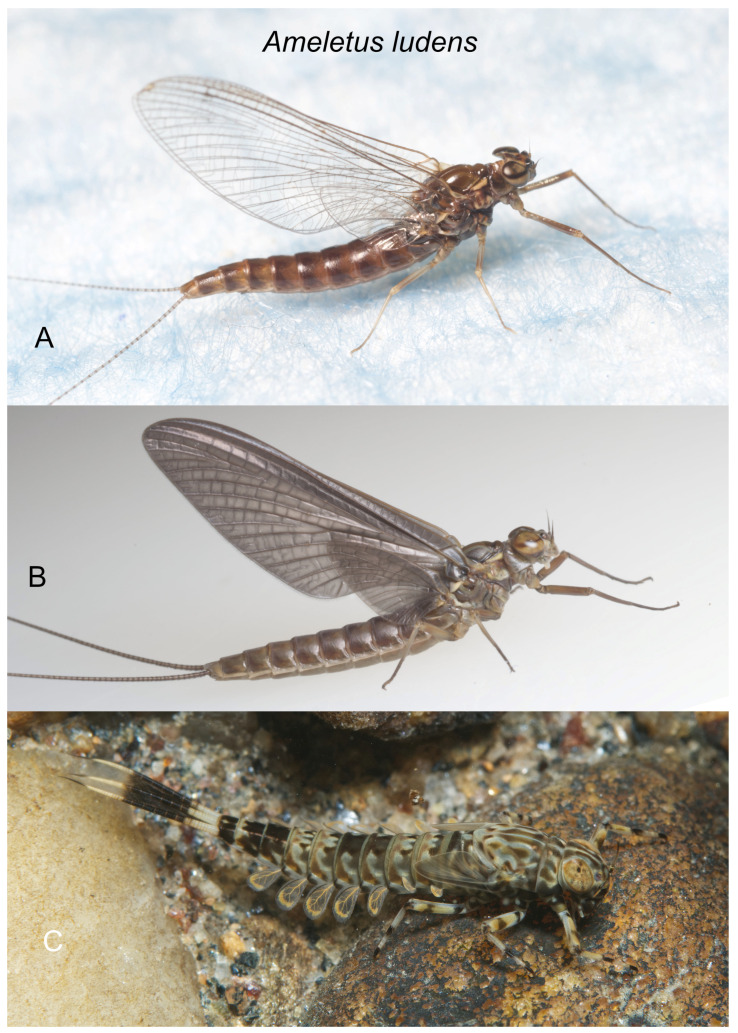
*Ameletus ludens*. (**A**). Living female imago, lateral view. (**B**). Living female subimago, lateral view (**C**). Living female larva, dorsolateral view.

**Figure 9:**
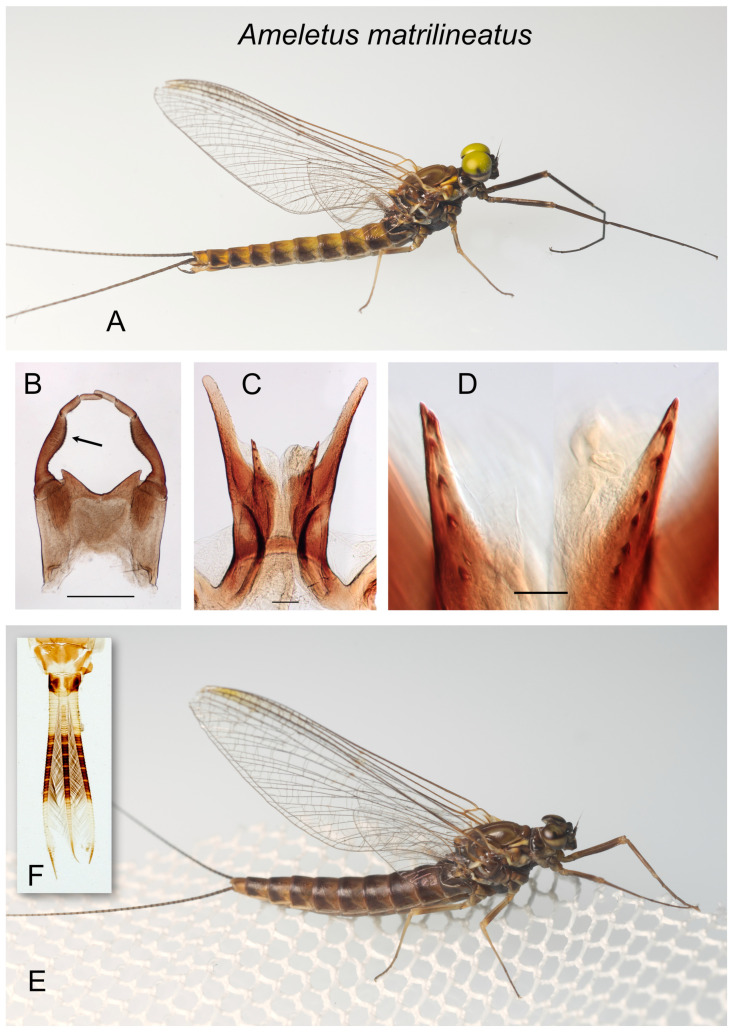
*Ameletus matrilineatus*. (**A**). Living male imago, lateral view. (**B**). Male styliger plate and forceps, dorsal view. Arrow indicates dilation of the long joint. Scale = 0.5 mm. (**C**). Penes of male, ventral view. Scale = 0.1 mm. (**D**). Ventral plates of penes, ventral view. Scale = 0.05 mm. (**E**). Living female imago, lateral view. (**F**). Caudal filaments of larva showing light speckling within the dark band on the middle third.

**Figure 10:**
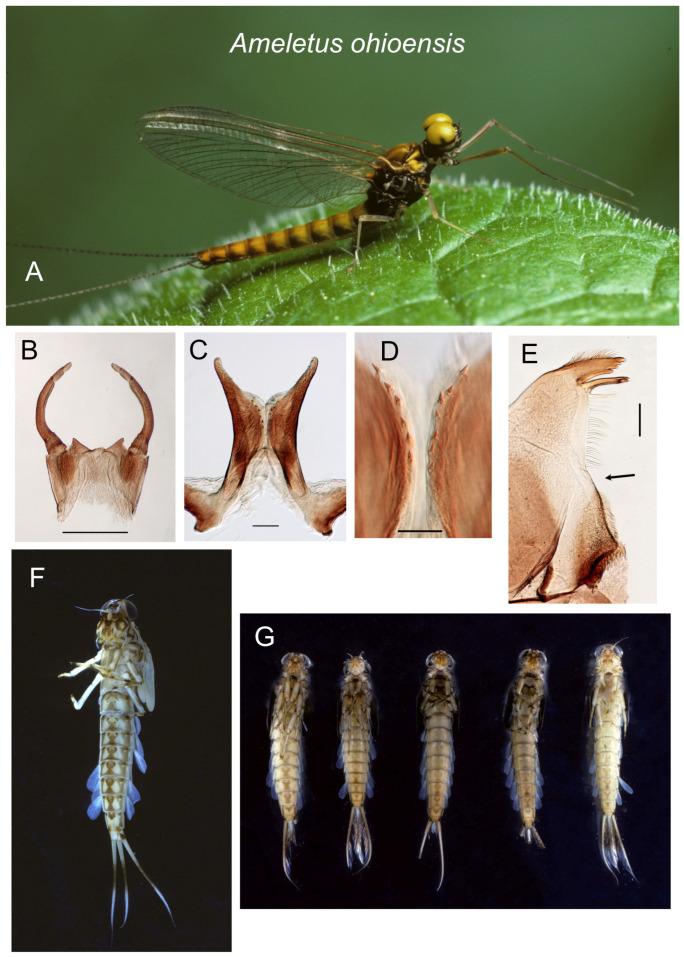
*Ameletus ohioensis*. (**A**). Living male imago, lateral view. (**B**). Male styliger plate and forceps, dorsal view. Scale = 0.5 mm. (**C**). Penes of male, ventral view. Scale = 0.1 mm. (**D**). Ventral plates of penes, ventral view. Scale = 0.05 mm. (**E**). Left mandible of larva, dorsal view. Arrow indicates setal gap. Scale = 0.1 mm. (**F**). Full-grown male larva, ventral view. (**G**). Full-grown male larvae, ventral view, showing variation in sternal color pattern.

**Figure 11:**
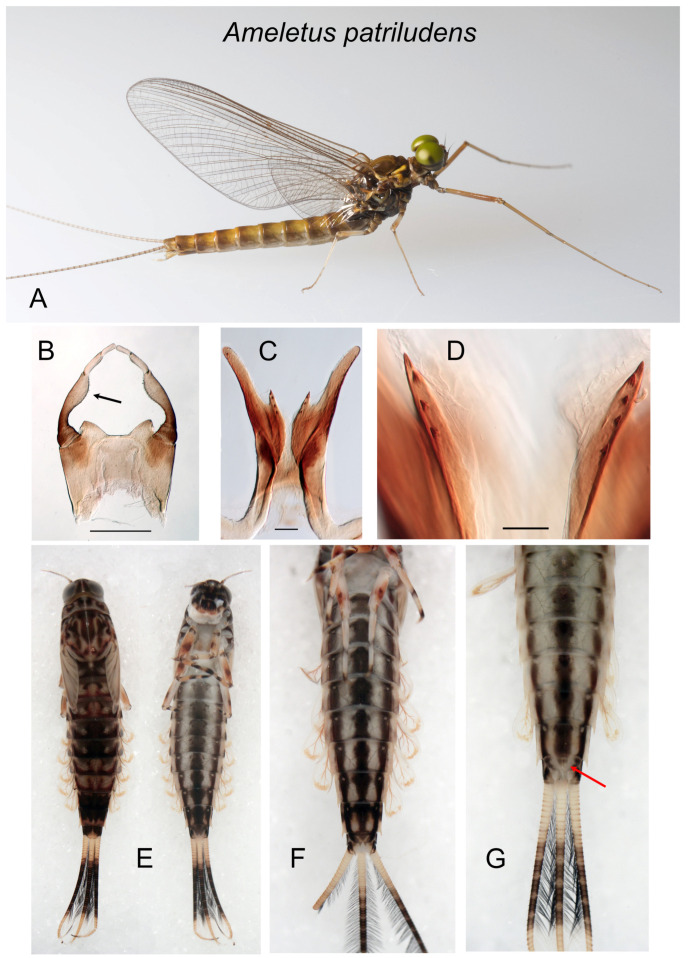
*Ameletus patriludens*. (**A**). Living male imago, lateral view. (**B**). Male styliger plate and forceps, dorsal view. Arrow indicates dilation of long joint. Scale = 0.5 mm. (**C**). Penes of male, ventral view. Scale = 0.1 mm. (**D**). Ventral plates of penes, ventral view. Scale = 0.05 mm. (**E**). Male larva, dorsal (left) and ventral views (**F**). Full-grown male larva, ventral view. (**G**). Full-grown male larva, ventral view. Color variant with ventral stripes that do not coalesce on sternite 9 (see Remarks in species account).

**Figure 12:**
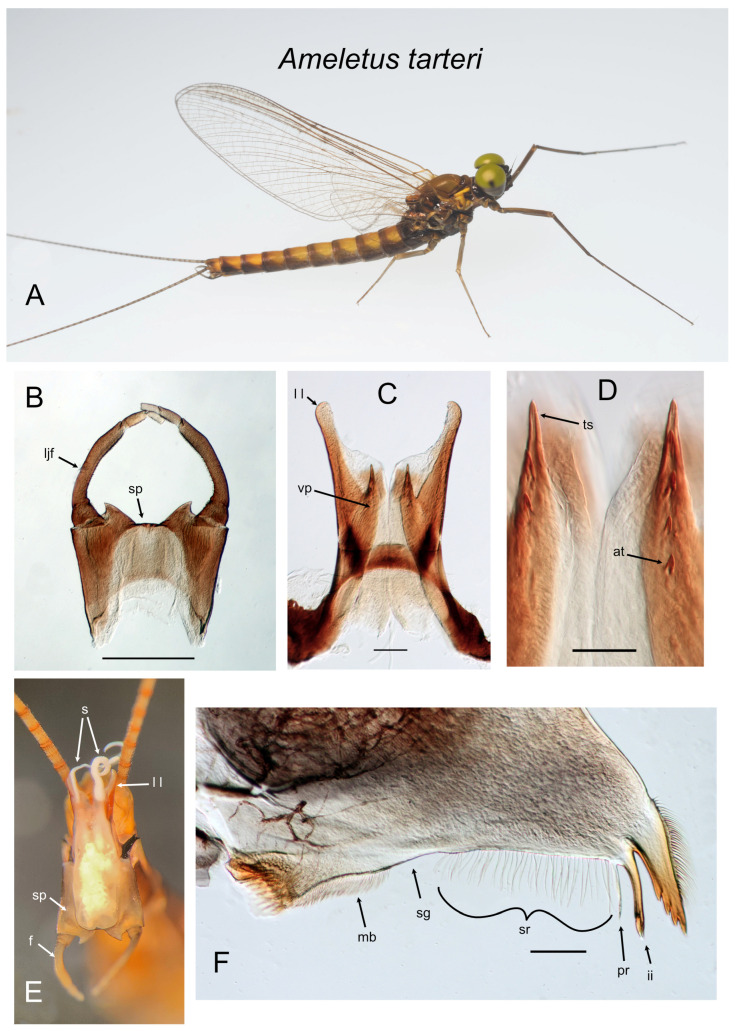
*Ameletus tarteri*. (**A**). Living male imago, lateral view. (**B**). Male styliger plate and forceps, dorsal view. Scale = 0.5 mm. (**C**). Penes of male, ventral view. Scale = 0.1 mm. (**D**). Ventral plates of penes, ventral view. Scale = 0.05 mm. (**E**). Male imago, distal end of abdomen in copulatory position, caudal view (**F**). Left mandible of larva, dorsal view. Scale = 0.1 mm.. Abbreviations: at—accessory tooth; f—forceps; ii—inner incisor; ljf—long joint of forceps; ll—lateral lobe of penes; mb—molar brush; pr—prostheca; s—sperm; sg—setal gap; sp—styliger plate; sr—setal row; ts—terminal spine of ventral plate; vp—ventral plate of penes.

**Figure 13:**
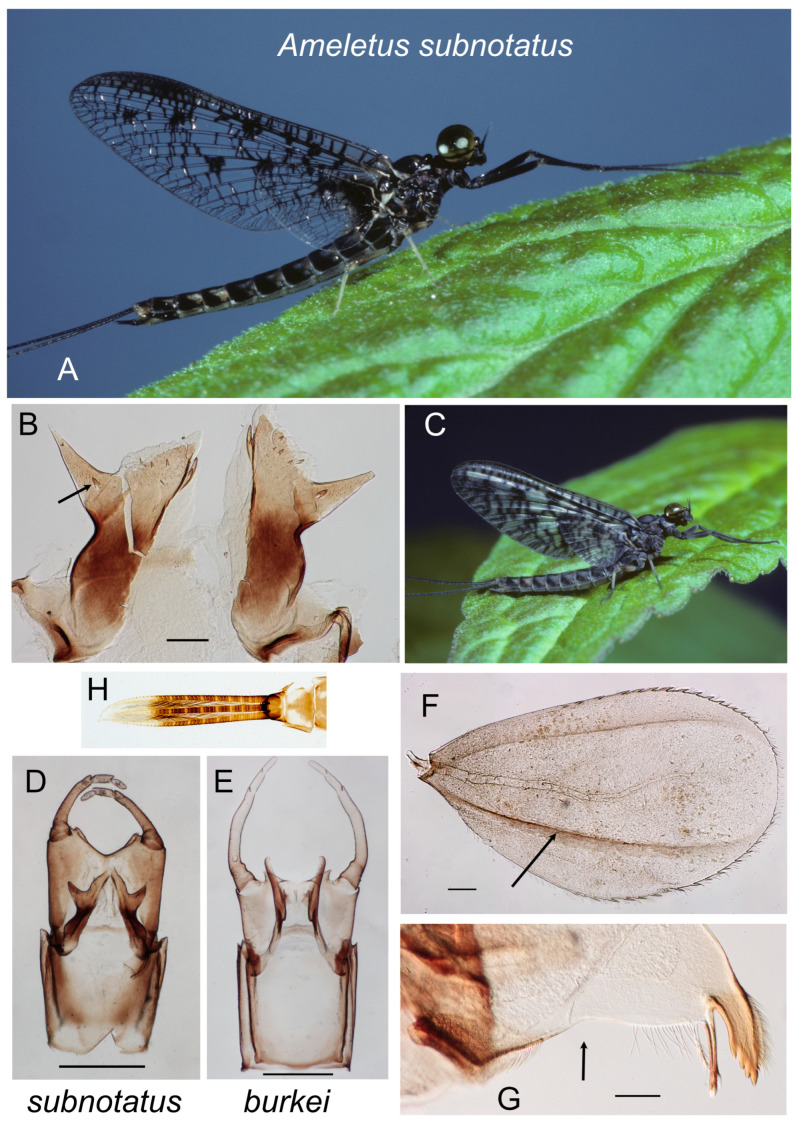
*Ameletus subnotatus* (except E: *burkei*). (**A**). Living male imago, lateral view. (**B**). Penes of male, ventral view. Arrow indicates tooth at base of lateral lobe. Scale = 0.1 mm. (**C**). Living male subimago, lateral view. (**D**). Male imago segment 9, penes, styliger plate and forceps (10th segment removed). Scale = 0.5 mm. (**E**). *A. burkei* male imago segment 9, penes, styliger plate and forceps (10th segment removed). Scale = 0.5 mm. (**F**). Larval gill 4 (right), dorsal view. Arrow indicates anal rib which is inset far from anal margin. Scale = 0.1 mm. (**G**). Left mandible of larva, dorsal view. Arrow indicates setal gap. Scale = 0.1 mm. (**H**). Caudal filaments of final larval instar (exuviae), dorsal view.

**Figure 14:**
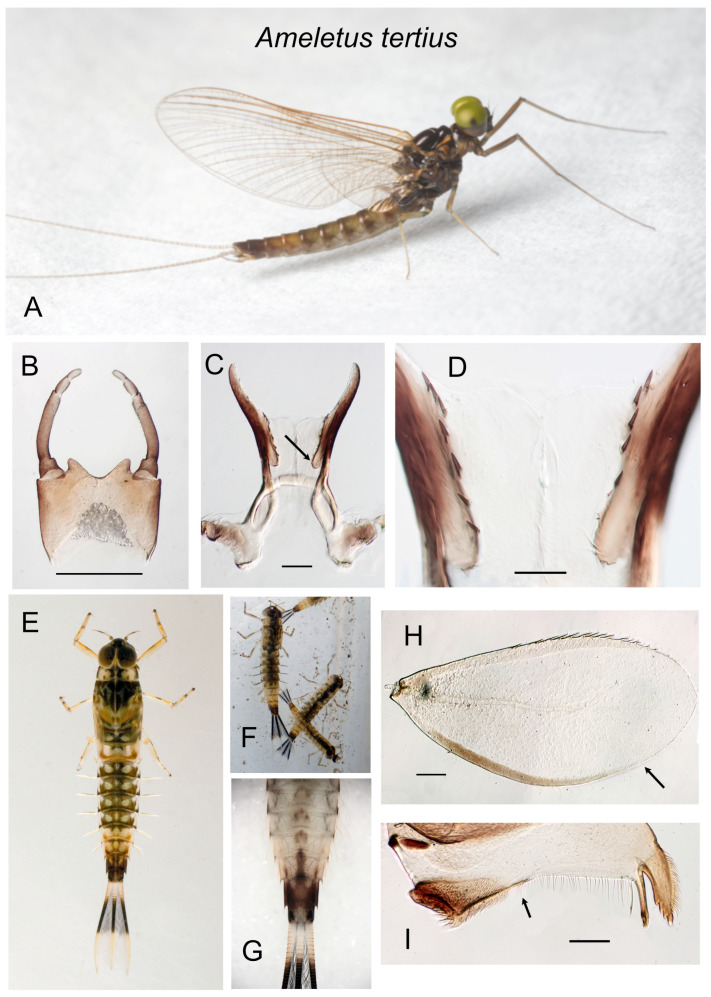
*Ameletus tertius*. (**A**). Living male imago, lateral view. (**B**). Male styliger plate and forceps, dorsal view. Scale = 0.5 mm. (**C**). Penes of male, ventral view. Arrow indicates the basal lobe of the ventral plate. Scale = 0.1 mm. (**D**). Ventral plates of penes, ventral view. Scale = 0.05 mm. (**E**). Living larva, dorsal view. (**F**). Living larvae of color morph with a dark band that extends to the base of the caudal filaments. (**G**). Larval abdomen, ventral view. (**H**). Larval gill 4 (right), dorsal view. Arrow indicates anal margin which lacks spines. Scale = 0.1 mm. (**I**). Left mandible of larva, dorsal view. Arrow indicates where the setal row is continuous with the molar brush, i.e., lack of setal gap. Scale = 0.1 mm.

**Figure 15:**
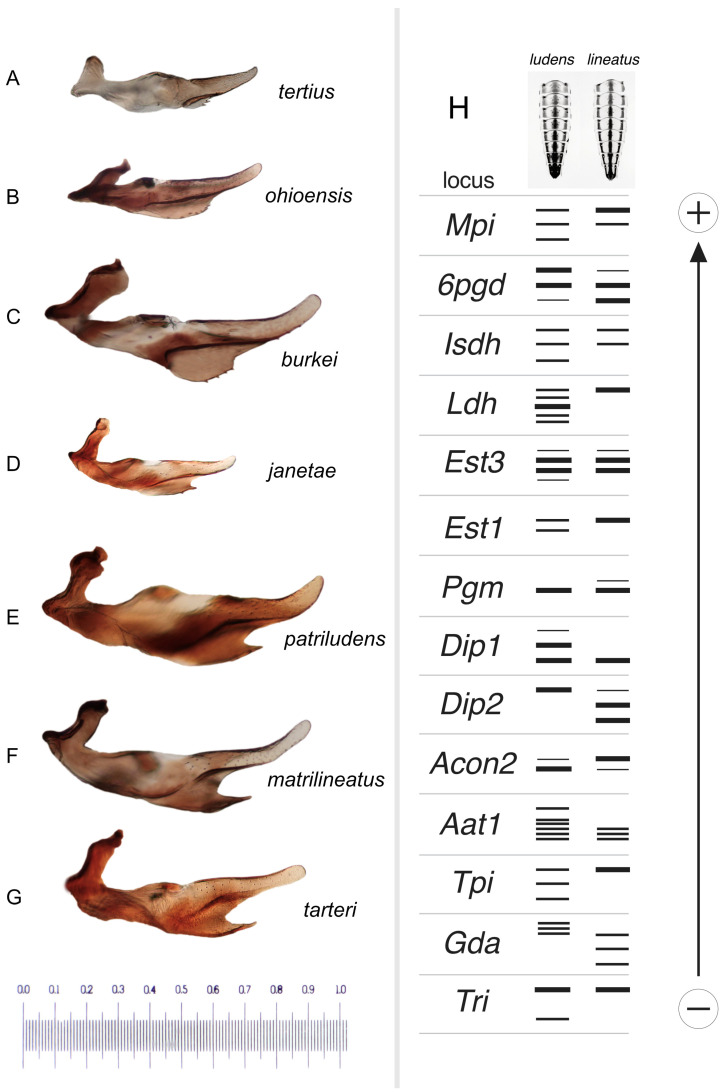
(**A**–**G**). Male penes, left lateral view, with species labeled. Scale in mm. (**H**). Graphic representation of allozyme banding patterns at 14 loci that distinguish the dominant *Ameletus ludens* and *A. lineatus* clones present in White Clay Creek, Pennsylvania, USA. Larval sternite color patterns above.

**Figure 16:**
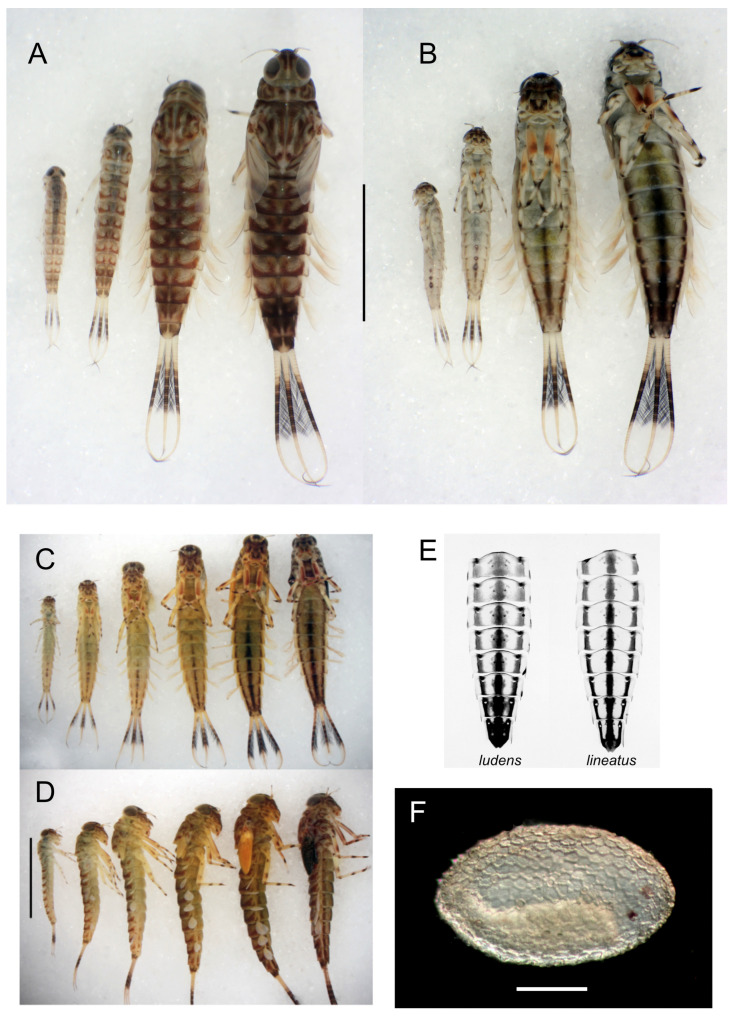
(**A**). *Ameletus ludens* larvae, clonal siblings, developmental series, dorsal view. Scale = 5 mm. (**B**). Same individuals as (**A**), ventral view. (**C**). *Ameletus matrilineatus* larvae, siblings, developmental series, ventral view. Scale = 5 mm. (**D**). Same individuals as (**C**), right lateral view. (**E**). *Ameletus ludens* and *lineatus*, sternites from final larval exuviae. (**F**). *Ameletus immaculatus* egg in post-diapause stage, with embryo visible. Scale = 0.1 mm.

**Figure 17:**
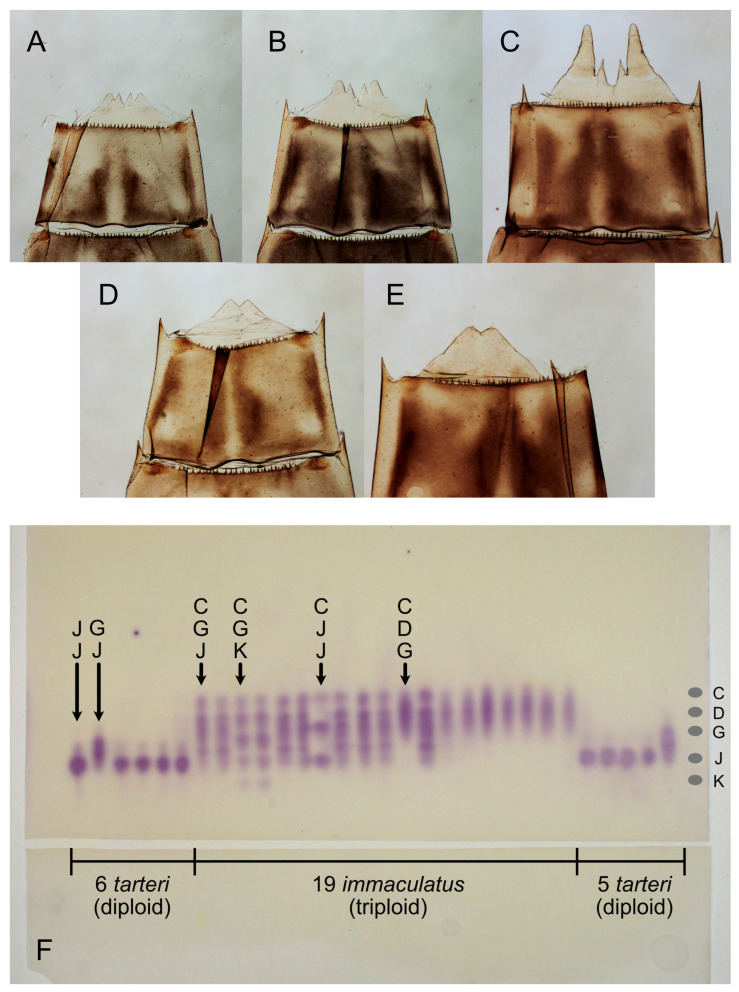
(**A**–**C**). Determining the gender of half- to full-grown larvae by the presence of developing styliger plate and forceps at the posterior margin of sternite 9 in male *Ameletus burkei* (larval exuviae with 10th segment removed for clarity). Body length (**A**) 6.5 mm; (**B**): 8.5 mm; (**C**): 12 mm (final larval instar). (**D**,**E**). Female larvae of *A. immaculatus* for comparison. Body length (**D**): 7 mm; (**E**): 12 mm. (**F**). Photograph of a starch gel stained for glucose phosphate isomerase (*Gpi*; E.C. 5.3.2.9). Homogenates from 30 individual adult mayflies (11 *A. tarteri* and 19 *A. immaculatus*) were electrophoresed (origins along the horizontal gap visible at the lower third of image). The five alleles present among these individuals (designated C, D, G, J, and K) are represented by gray elliptical spots on the right. Six distinct genotypes are labeled at the top. Nine individuals of *A. tarteri* were homozygous for allele J, resulting in a single band of activity. Two of the *A. tarteri* and all 19 of the *A. immaculatus* were heterozygous. *Gpi* is dimeric (composed of two subunits) such that a diploid individual, which is heterozygous for two alleles, will stain with three bands: a slower band corresponding to one allele, a faster band from the other allele, and a hybrid band with intermediate mobility. If the polypeptides produced by the two alleles contribute equally and the subunits combine randomly, the relative intensity of the three bands will be 1:2:1 (slow:hybrid:fast, e.g., genotype GJ). In the case of triploids, if an individual is heterozygous with three different alleles, there will be 6 bands (e.g., genotype CGK), although depending on the relative mobilities of the subunits, hybrid bands may superimpose on primary bands. Triploids with two copies of one allele and one of the second allele will show three bands (e.g., CJJ) with relative intensities of 4:4:1.

### 3.4. Taxonomic Treatment


 
***Ameletus browni* species group**
 


The *A. browni* group is proposed here and includes *Ameletus browni*, *A. rebucki*, and *A. cryptostimulus*. This group is endemic to the eastern Nearctic. Adult males share a distinctive recurved (U-shaped) structure on the ventral plates of the penes (Figure 1C, Figure 2C and Figure 3C) unique to this group. Larvae can be distinguished from other eastern Nearctic *Ameletus* by gill 4, which has the anal rib on its margin (i.e., not inset) and both the costal and anal margins have robust spines.


 
***Ameletus browni* McDunnough**
(Figure 1A–F) 


*Ameletus browni* McDunnough [12]: 278; Needham et al. [13]: 450; Zloty [1]: 303.


 


**Type Material.** Male holotype and female allotype: Mt.Lyall, Gaspe County, Quebec, Canada 11 August 1933; No. 3652 in CNC (male paratype 3652 examined).


 


**Diagnosis**. *Ameletus browni* is a diploid bisexual species. Male imagos (Figure 1A) are clear-winged with a slight amber tinge and light brown veins and cross-veins. Upper portion of eyes yellow-green in life. They can be distinguished from most other eastern Nearctic species by the ventral plates of penes which form a distinctive U-shaped structure, the central portion consisting of a single large spine without accessory teeth or spinules (Figure 1C,D). *Ameletus cryptostimulus* and *A. rebucki* are similar in this regard, but *A. browni* can be distinguished from those by the shape of the basal sclerite of the penes (arrow in Figure 1C) and the relatively short and slender lateral lobes.

Larvae can be distinguished from all other eastern Nearctic species by the following combination: wide setal gap on the mandible (Figure 1E); gill 4 with spines on the costal and anal margins and with anal rib on the margin (i.e., not inset; as in Figure 3G); dark band on caudal filaments evenly colored and extending from near base to about 2/3 out to tips (Figure 1F); dorsum of abdomen with color pattern forming a pale median longitudinal stripe; spines present on posterior margins of all tergites.


 


**Distribution.** Quebec and New England south to northern Pennsylvania.


 


**Phenology.** Apparently univoltine, with emergence in mid-May in the southern portion of its range to mid-August in the north. Emergence begins later in the season than species with which it is commonly found (*A. rebucki*, *A. ludens*, *A. immaculatus*). Egg development has not been documented.


 


**Remarks**. *Ameletus browni* larvae can usually be spotted easily in the field by the distinctive pale median stripe running down the terga. Similarities to both *A. cryptostimulus* and *A. rebucki* with regard to the gills of larvae and the ventral plate of the penes in adult males, as well as genetic data (both COI and allozymes), show the close relationship among these three. *Ameletus browni* can often be found together with *A. rebucki*. The latter lacks the dorsal stripe in the larva, is larger, and emerges earlier in the year.


 
***Ameletus cryptostimulus* Carle**
(Figure 2A–F) 


*Ameletus cryptostimulus* Carle [14]: 581; Zloty [1]: 305.


 


**Type Material.** Male holotype and female allotype: Little Stony Creek, Giles County, Virginia, USA 10 April 1977, F. Carle; in USNM (not examined).


 


**Diagnosis**. *Ameletus cryptostimulus* is a diploid bisexual species. Male imagos (Figure 2A) are clear-winged with a very slight amber tinge and light brown veins and cross-veins. The body size ranges from 9 to 11 mm, forewing length 9–11 mm. Upper portion of eyes yellow to green in life. They can be distinguished from most other eastern Nearctic species by the ventral plates of penes, which form a distinctive U-shaped structure, the central portion consisting of a single large spine without accessory teeth or spinules (Figure 2C,D). *Ameletus browni* and *A. rebucki* are similar in this regard, but *A. cryptostimulus* can be distinguished from *A. browni* by the shape of the basal sclerite of the penes (compare the arrow in Figure 1C with Figure 2C) and the relatively long and wide lateral lobes. Compared to *A. rebucki*, the lateral lobes of the penes in *A. cryptostimulus* are shorter relative to the U-shaped ventral plates (Figure 2C vs. Figure 3C), and *A. cryptostimulus* males are more brightly colored (see Figure 2A vs. Figure 3A).

Larvae can be distinguished from all other eastern Nearctic species by the following combination: wide setal gap on the mandible that is about as long as setal row (Figure 2E); gill 4 with spines on the costal and anal margins and anal rib on margin (i.e., not inset; as in Figure 3G); dark band on caudal filaments evenly colored and extending from near base to about 2/3 out to tips; dorsum of abdomen without a pale median longitudinal stripe; spines present on posterior margins of tergites 5– or 6–10 (missing on 1–4).


 


**Distribution**. Mountains of Virginia, North Carolina, South Carolina, and eastern Tennessee.


 


**Phenology**. Univoltine, with emergence from March through June (Huryn and Wallace [15]; predominantly April and May). Eggs begin hatching in summer (without a diapause), but hatching continues over an extended period into autumn.


 


**Remarks**. *Ameletus cryptostimulus*, *A. rebucki*, and *A. browni* form a distinct group (the *browni* group) based on male genitalia and larval gills. Morphologically, *A. cryptostimulus* is most similar to *A. rebucki*, but the two appear to have non-overlapping ranges.


 
***Ameletus rebucki* sp. nov.**
(Figure 3A–G) 


*Ameletus cryptostimulus* Carle; Zloty [1]: 305 (in part).


 


**Description.** Male holotype imago (fresh, in alcohol) (Figure 3A).

Body length 9 mm, forewing length 9.5 mm. Head blackish-brown; compound eyes dark greenish-brown dorsally, black laterally. Prothoracic tergum dark brown; mesotergum brown with slightly paler submedian streaks, scutellum blackish-brown; metatergum dark brown; thoracic pleuron dark brown with paler areas at the base of wings, legs, and surrounding mesothoracic spiracle. Fore legs dark brown; mid- and hind legs light brown. Wings transparent with dark infuscation in stigmatic area. Longitudinal veins mostly dark brown (lighter toward costal region), cross-veins dark brown. Abdominal tergite 1 dark brown; tergites 2–8 dark creamy with dark brown shading on posterior margin that extends medially and laterally to form triangular posterior lateral patches; tergite 9–10 dark brown. Abdominal sternite 1 brown; sternites 2–8 creamy with brown submedian spots and distinct ganglionic spots; 9 pale creamy with dark brown laterally. Styliger plate creamy; forceps brown. Caudal filaments uniformly light brown. Long joint of forceps with uniform thickness through its length (Figure 3B). Penes (Figure 3C) with ventral plates produced into a distinctive U-shaped structure, with a large median spine and no accessory teeth (Figure 3D).

Larva: Body length 10–13 mm [holotype 10 mm]. Dorsally with pale median and lateral spots on a dark brown base (Figure 3E). Sternal coloration mostly pale, often distinctly darkened on the lateral ¼ of each sternite. Mandible with a wide setal gap as long or longer than setal row (Figure 3F). Legs mostly brown, not distinctly patterned except for black-tipped tarsi. Small spines present on posterior margins of terga 3– or 4–10. Gill 4 of mature larvae with anal rib on anal margin and spines present on both costal and anal margins (Figure 3G). Caudal filaments usually dark from base but with a darker band in middle 1/3 (with all articulations similarly colored) and with distal 1/3 pale.


 


**Type Material.** Male imago holotype. Reared from larva, tributary of Wyalusing Creek, 4.8 km W of Montrose, Susquehanna County, Pennsylvania, USA (41.81972° N, 75.93333° W, elev. 372 m), 24 April 1985, D.I.Rebuck and D.H.Funk. One female allotype and two male paratypes, reared from larvae 26 April 1984, same data. Types will be deposited at PERC.


 


**Etymology.** Named for David I. Rebuck, whose meticulous field and laboratory work helped make this study possible.


 


**Diagnosis**. *Ameletus rebucki* is a diploid bisexual species. Adult males can be distinguished from most other eastern Nearctic species by the ventral plates of penes which form a distinctive U-shaped structure, the central portion consisting of a single large spine without accessory teeth or spinules (Figure 3C,D). *Ameletus browni* and *A. cryptostimulus* have similar ventral plates, but *A. rebucki* can be distinguished from *A. browni* by the shape of the basal sclerite of the penes (see arrow in Figure 1C) and the relatively long and wide lateral lobes. Compared to *A. cryptostimulus*, the lateral lobes of the penes in *A. rebucki* are longer relative to the U-shaped ventral plates (Figure 3C vs. Figure 2C), and *A. rebucki* males are darker than *A. cryptostimulus* (see Figure 3A). Also, the upper portion of the compound eyes is dark brown in life, with usually only a hint of greenish.

Larvae can be distinguished from all other eastern Nearctic species by the following combination of characters: wide setal gap on the mandible that is subequal to the length of the setal row (Figure 3F); gill 4 with spines on the costal and anal margins and with anal rib on margin (i.e., not inset; Figure 3G); caudal filaments usually dark from base, with a darker, evenly colored band in middle 2/3 and paler beyond (Figure 3E); dorsum of abdomen dark with paler spots, but without a pale median longitudinal stripe; spines present on posterior margins of tergites 3– or 4–10 (missing on 1–2 or –3).


 


**Distribution**. Vermont and New Hampshire south to West Virginia.


 


**Phenology**. *Ameletus rebucki* is univoltine but does not have a summer egg diapause. Emergence is early, beginning in late March in West Virginia and Pennsylvania to late April in Vermont.


 


**Remarks**. Previous records of *A. cryptostimulus* from areas north of Virginia are attributable to *A. rebucki*. See remarks under *A. cryptostimulus*.


 
***Ameletus ludens* species group**
 


The *A. ludens* group is proposed here and includes *A. burkei*, *A. immaculatus*, *A. janetae*, *A. lineatus*, *A. ludens*, *A. matrilineatus*, *A. patriludens*, *A. ohioensis*, and *A. tarteri*. It includes the three known polyploid parthenogens and their bisexual progenitors. Adult males can be distinguished from other eastern Nearctic *Ameletus* (except for *A. tertius*) by their long lateral lobes (reaching beyond the forceps base; Figure 13E vs. Figure 13D) and by the ventral plate not being recurved (as in *A. browni* group). Larvae are the only eastern Nearctic species with the anal rib of gill 4 narrowly inset (as in Figure 5E).


 
***Ameletus burkei* sp. nov.**
(Figure 4A–G, Figure 13E, Figure 15C and Figure 17A–C) 


**Description.** Male holotype imago (fresh, in alcohol) (Figure 4A). Body length 13 mm, forewing length 12 mm. Head blackish-brown with paler patches on face and between compound eyes and antennae; compound eyes pale yellow-green dorsally with a black line laterally, light gray immediately below, then dark gray below that. Prothoracic tergum blackish-brown; mesotergum light brown, scutellum light brown changing to blackish at the posterior lateral areas; metatergum blackish; thoracic pleuron dark brown with paler areas at the base of wings, legs, and surrounding mesothoracic spiracle. Forelegs gray-brown; mid- and hind legs pale creamy. Wings transparent. Longitudinal veins amber brown, cross-veins light brown. Abdominal tergite 1 dark brown; tergites 2–9 yellowish with brown shading on posterior margin that extends and forms triangular posterior lateral patches; tergite 10 yellowish with brown submedian streaks. Abdominal sternites 3–7 with conspicuous ganglionic markings; sternite 1 blackish; sternites 2–4 creamy yellowish with brown submedian spots; 5–8 yellowish; 9 yellowish with dark brown along lateral margin. Styliger plate creamy yellowish; basal half of forceps long joint gray brown, distally yellow to creamy. Caudal filaments light brown with a reddish-brown ring near the base of each segment. Long joint of forceps of uniform thickness through its length (Figure 4B). Penes (Figure 4B and Figure 15C) with broad ventral plates, not produced into a spine posteriorly, with a row of 3–6 teeth along the ventral margin (Figure 4D).

Larva: (Figure 4E). Body length 10–16 mm (holotype 13). Dorsal color pattern subtle and variable. Sternal coloration also variable, but usually pale creamy with two pairs of small submedian dots on most sternites, frequently with dark shading near lateral margins on 1–9, which sometimes extends across the sternite. Mandible with distinct setal gap, 20–40% length of setal row (Figure 4F). Legs not distinctly patterned except for apical black band on tarsi and white patch on the distal 1/3 of the anterior face of the femora, the latter visible in living specimens. Small spines always present on the posterior margins of terga 4–10; most individuals also have smaller spines on tergum 3, and often traces on tergum 2. Gill 4 of mature larvae with anal rib narrowly inset, with spines present on both costal and anal margins (as in Figure 5E). Caudal filaments pale at base with a distinct dark band in middle 1/3 (with all articulations similarly colored) and with distal tips darkened.


 


**Type Material.** Male imago holotype. Reared from larva, headwaters of Station Spring Creek, Tazewell County, Virginia, USA (37.087180° N, 81.403560° W, elev. 1100 m), 15 April 2024, D.H.Funk and A.C.Graham. Three male, one female imago paratypes, reared from larvae, same locality, 22 April 1990, D.H.Funk. Types will be deposited at PERC.


 


**Etymology.** Named for the type locality in Burke’s Garden, Virginia.


 


**Diagnosis.** *Ameletus burkei* is a diploid bisexual species. Adult males can be distinguished from all other eastern Nearctic *Ameletus* by the following combination of characters: long lateral lobes of the penes extending beyond the base of the forceps; long joint of forceps with uniform thickness (i.e., without dilation in apical 1/3); ventral plates of the penes neither produced into a spine distally, nor with a prominent rounded lobe projecting anteriorly, but extending ventrally into a broad plate with 2–6 teeth along its rounded ventral margin (Figure 15C). Larvae can be distinguished from most other eastern Nearctic species by the following combination: distinct but narrow setal gap on the mandible; gill 4 with spines on both the costal and anal margins, with anal rib narrowly inset (as in Figure 5E); dark band in middle 1/3 of caudal filaments with segments uniformly colored; venter of abdomen without distinct stripes (Figure 4E). *Ameletus burkei* larvae are not distinguishable from *A. immaculatus* other than by the presence of males, and from *A. tarteri* or *A. ohioensis* other than known geographic range or COI haplotype.


 


**Distribution.** Presently known only from the type locality in southwest Virginia.


 


**Phenology.** *Ameletus burkei* is univoltine, with an obligate summer egg diapause. Adults are present in mid-April and emergence likely extends through May.


 


**Remarks.** Genetic data indicate *A. burkei* is the closest known relative to the maternal ancestor of the obligate thelytokous parthenogen *A. immaculatus*, with which it coexists at the type locality in Station Spring Creek. There, *A. burkei* (and *A. immaculatus*) are found in the headwaters and are replaced by *A. matrilineatus* downstream in the same pattern evident in much of the eastern U.S., where the unstriped parthenogen *A. immaculatus* is found in headwaters and is replaced by one or both of the striped parthenogens *A. ludens* and *A. lineatus* downstream.

*Ameletus burkei* is presently known only from the type locality. Future collections may reveal that the species is more widespread. However, as discussed under *A. matrilineatus* (below), the apparent uniqueness of the Station Spring Creek fauna may be due to its physical isolation.


 
***Ameletus immaculatus* sp. nov.**
(Figure 5A–E, Figure 16F and Figure 17D,E) 


**Description.** Female holotype imago (fresh, in alcohol) (Figure 5A). Body length 10 mm, forewing length 10.5 mm. Head brown with paler patches on face and between compound eyes and antennae, and on vertex; compound eyes dark gray dorsally with a black line laterally, light gray in middle 1/3, black again below. Prothoracic tergum brown; mesotergum yellowish brown, scutellum light brown changing to black at the posterior lateral areas; metatergum blackish; thoracic pleuron dark brown with paler areas at the base of wings, legs, and surrounding mesothoracic spiracle. All legs light brown. Wings transparent. Longitudinal veins and cross-veins brown. Abdominal tergite 1 dark brown; tergites 2–9 light brown with darker shading on posterior margin that extends and forms triangular posterior lateral patches; tergite 10 light brown with dark brown submedian streaks. Abdominal sternites without conspicuous ganglionic markings; sternite 1 brown; sternites 2–9 light red-brown. Caudal filaments light brown with a dark ring near the base of each segment.

Larva: Body length 8–16 mm. Dorsal color pattern variable but subtle (Figure 5B,C). Sterna unpatterned except for two pairs of small submedian dots on most sternites, and sometimes dark shading near lateral margins of sternum 9, which may extend anteriorly to 8 and 7. Mandible with distinct setal gap, 20–40% length of setal row (Figure 5D). Legs not distinctly patterned except for apical black band on tarsi and white patch on the distal 1/3 of the anterior face of the femora, the latter visible in living specimens. Small spines always present on posterior margins of terga 4–10 in full-grown larvae; larger individuals also have smaller spines on tergum 3, and often traces on tergum 2. Gill 4 of full-grown larvae with anal rib narrowly inset, and spines present on both costal and anal margins (Figure 5E). Caudal filaments pale at base with a distinct dark band in middle 1/3 (with all articulations similarly colored) and with distal tips darkened.


 


**Type Material.** Female imago holotype. Reared from larva, headwaters of Puncheoncamp Branch, Browns Creek, McDowell County, West Virginia, USA (37.486243° N, 81.565984° W, elev. 563 m), 22 April 1990, D.H.Funk. Two female imago paratypes, same data. Types will be deposited at PERC.


 


**Etymology.** *immaculatus* = unmarked (Latin), in reference to the lack of ventral stripes in the larva, which are present in the other eastern Nearctic parthenogens.


 


**Diagnosis.** *Ameletus immaculatus* is a clonal, obligate thelytokous parthenogen. All clones that have been tested are triploid. Adults are variable in size, with body length up to 14 mm and wing length up to 15 mm. Size not only decreases through the emergence period but also varies considerably by population. The female imago is predominantly dark brown or reddish brown, with clear wings and brown venation, and aside from the absence of ganglionic spots on the venter of the abdomen (which may be present to varying degrees in its close relatives), *A. immaculatus* is not distinguishable morphologically from females of other clear-winged species. The subimago looks similar but with a dull gray overcast and slate gray wings (as in Figure 7B). Full grown larvae range up to 16 mm body length but may be as small as 8 mm. *Ameletus immaculatus* larvae can be distinguished from most other eastern Nearctic species by the combination of the presence of a narrow but distinct setal gap in the mandible; a distinct (but uniformly colored, i.e., unspeckled) dark band in the middle 1/3 of the caudal filaments; anal rib narrowly inset on gill 4; the lack of dark patterning on the venter of the abdomen (other than small submedian dots and some shading on the lateral margins of sternite 8 and 9); and the absence of males. However, the only sure way to distinguish *A. immaculatus* from female larvae of the bisexual species *A. burkei*, *A. tarteri*, and *A. ohioensis*, with which it may co-occur, is by ploidy level, parthenogenetic reproductive mode, and COI haplotype.


 


**Distribution.** From northern New England south to North Carolina, west to Ohio.


 


**Phenology.** *Ameletus immaculatus* is univoltine, with an obligate summer egg diapause. Young larvae first appear in autumn and grow through winter and early spring. Adults are present from mid-March in the south and at lower elevations, through June in more northern areas and higher elevations.


 


**Remarks.** *Ameletus immaculatus* is a widespread, but previously unrecognized, obligately parthenogenetic species. It inhabits small headwater streams and springs and is nearly always found in proximity to one or both of the other two eastern Nearctic parthenogens *A. ludens* and *A. lineatus* (in fact, Traver [16] may have been looking at an *A. immaculatus* imago when she determined that *A. lineatus* imagos lack ganglionic spots). In a given stream, the three parthenogens have an upstream-downstream distributional relationship, with *A. immaculatus* being the upstream member but mixing with one or both of the others in the middle reaches, and *A. immaculatus* being absent downstream. As is the case with *A. ludens* and *A. lineatus*, *A. immaculatus* has a much broader geographic distribution than any of its close bisexual relatives (viz., *A. burkei*, *A. ohioensis*, or *A. tarteri*). At sites where these bisexuals are found, *A. immaculatus* is usually also present, and larvae are difficult or impossible to distinguish from those except by their sexuality, ploidy level, or COI haplotype. Published larval records of *A. tarteri* from localities other than eastern West Virginia likely represent misidentified *A. immaculatus*.

The type locality of *A. immaculatus* in southern West Virginia is now, like many first-order streams in that region, buried beneath valley fill from a nearby coal mining operation.


 
***Ameletus janetae* Kondratieff and Meyer**
(Figure 6A–G and Figure 15D) 


*Ameletus janetae* Kondratieff and Meyer [2]: 526.


 


**Type Material.** Male imago holotype. Hardy County, West Virginia, USA 3mi NE [sic] Mathias, 38.917° N, 78.883° W, 8–29 May 2008, D. R. Smith, Malaise Trap (USNM). Paratype: 1 male, same data as holotype (CSUC).


 


**Diagnosis.** *Ameletus janetae* is a diploid bisexual species. Male imagos (Figure 6A) can be distinguished from other eastern Nearctic *Ameletus* species by the following combination of characters: long lateral lobes of the penes extending beyond the base of the forceps; long joint of forceps with slight dilation in the apical 1/2 (Kondratieff and Meyer [2]: Figure 1; Figure 6B); ventral plates of the penes produced into a very small spine distally (Kondratieff and Meyer [2]: Figure 3; Figure 15D), with 3–5 teeth along their ventral margins (Figure 6D).

Larvae can be distinguished from all other eastern Nearctic species by the following combination: distinct but narrow setal gap on the mandible (Figure 6E); gill 4 with spines on both the costal and anal margins, with anal rib narrowly inset (as in Figure 5E); dark band in middle portion of caudal filaments with every 4th segment pale, giving it a speckled appearance (Figure 6F–H); venter of abdomen with three dark stripes (median stripe often broken into a series of spots) that do not coalesce on sternite 9 (Figure 6H); spines present on posterior margins of tergites 4–9 in late instars (sometimes small and scattered on 3, but absent from 1 and 2).


 


**Distribution.** Known only from a few streams in the vicinity of the type locality in eastern West Virginia.


 


**Phenology.** Presumably univoltine, with a summer egg diapause, but the latter is undocumented. Emergence in May. All streams where the larvae have been collected had no surface flow in late summer and early autumn.


 


**Remarks.** Kondratieff and Meyer [2] described *A. janetae* from two male imagos captured in a malaise trap near Lost River State Park, NW of Mathias, West Virginia. I was able to rear larvae from three first-order tributaries to Howard’s Lick Creek near the type locality in 2022 through 2024. All three of those streams were dry at the surface from late July through early October. No *A. janetae* larvae were found in the mainstem of Howard’s Lick Creek, which retained surface flow throughout the year. *Ameletus immaculatus* was abundant in the streams where *A. janetae* was found and vastly outnumbered the latter. *Ameletus ludens* and *A. lineatus* were common just downstream of these sites.


 
***Ameletus lineatus* Traver**
(Figure 7A–D, Figure 15H and Figure 16E) 


*Ameletus lineatus* Traver [16]: 194; Traver [13]: 453; Burks [17]:102.


 


**Type Material.** Female imago holotype. Reared from nymph, Big Alamance Creek, North Carolina, USA, Feb. 20, 1930. No. 1079.1 in CUIC. Two female subimago paratypes, same data Feb. 28, 1930. No. 1079.2-3 CUIC.


 


**Diagnosis.** *Ameletus lineatus* is a clonal, obligate thelytokous parthenogen. All clones that have been tested are triploid. Adults are quite variable in size; wing length of large individuals 13 mm, but the size drops off dramatically through the emergence period (see Figure 21). The female imago is predominantly dark brown or reddish brown, with clear wings and brown venation (Figure 7A), and like *A. ludens*, is not distinguishable morphologically from females of other clear-winged species. The subimago looks similar but with a dull gray overcast and slate gray wings (Figure 7B). Full grown larvae range up to 14.5 mm body length and are distinguished from all eastern Nearctic species (except for *A. matrilineatus*) by the following combination of characters: the presence of a narrow setal gap on the mandible (Figure 7C); caudal filaments with a distinct dark band in middle 1/2, with every 4th segment within band lighter, giving the band a speckled appearance (as in Figure 9F); small spines present on posterior margins of terga 1–10 (may be quite small on tergum 1, but always distinct on 2–10 in full-grown larvae); abdomen ventrally with three narrow dark stripes with distinct margins that coalesce at the posterior margin of sternum 9 (Figure 16E). Distinguishable from *matrilineatus* only by the absence of males (see Figure 17A–E) and ploidy level.


 


**Distribution.** From Pennsylvania south to South Carolina, west to Missouri and Arkansas.


 


**Phenology.** *Ameletus lineatus* is univoltine, with an obligate summer egg diapause. Young larvae first appear in autumn and grow through winter and early spring. Adults are present from mid-March in the south and at lower elevations, through May in more northern areas and higher elevations.


 


**Remarks.** Traver [16] described *A. lineatus* from adult females reared from larvae collected from the piedmont of North Carolina.

See remarks under *A. ludens* (below). Because ventral stripes on larvae do not completely develop until the larva is fully grown (see Figure 17C–D), the distinction between *A. ludens* and *A. lineatus* (with which it commonly occurs) is not possible for younger larvae. The range of *A. lineatus* extends further south and west than *A. ludens* and because of the difficulty in distinguishing the two morphologically, I consider published records of *A. lineatus* from north of Pennsylvania, Ohio, Indiana and Illinois to be questionable.


 
***Ameletus ludens* Needham**
(Figure 8A–C and Figure 16A,B,E) 


*Ameletus ludens* Needham [18]: 36; Morgan [19]: 117; Needham [20]: 310; Traver [18]: 454; Traver [16]: 194; Burks [17]:103.


 


**Type Material.** None designated but described from larvae and female subimagos collected at Newport, New York, USA (CUIC) (examined).


 


**Diagnosis.** *Ameletus ludens* is a clonal, obligate thelytokous parthenogen. All clones that have been tested are triploid. Adults are quite variable in size; wing length of large individuals 12.5 mm, but the size drops off dramatically through the emergence period (see Figure 21). The female imago is predominantly dark brown or reddish brown, with clear wings and brown venation (Figure 8A), and is not distinguishable morphologically from females of other clear-winged species. The subimago looks similar but with a dull gray overcast and slate gray wings (Figure 8B). Full-grown larvae (Figure 8C) range up to about 13 mm body length and are distinguished from all eastern Nearctic species (except for *A. patriludens*) by the following combination of characters: the presence of a narrow setal gap on the mandible (as in Figure 7C); caudal filaments with a distinct dark band in middle 1/2, with every 4th segment within band lighter, giving the band a speckled appearance (as in Figure 9F); small spines present on posterior margins of terga 1–10 (may be quite small on tergum 1, but always distinct on 2–10 in full-grown larvae); abdomen ventrally with three dark stripes that coalesce on sternum 9 (and to varying degrees on 7 and 8), with the median stripe wider and its borders poorly defined compared with those of *A. lineatus* and *A. matrilineatus* (Figure 16B,E). Distinguishable from *patriludens* only by the absence of males (see Figure 17A–E), ploidy level, and COI haplotype.


 


**Distribution.** From eastern Canada (southern Ontario to Newfoundland) to the southern Appalachians; south of Pennsylvania generally restricted to the mountains.


 


**Phenology.** *Ameletus ludens* is univoltine, with an obligate summer egg diapause. Young larvae first appear in autumn and continue to grow through winter and early spring. Adults are present from early spring in the south and at lower elevations, through early summer in the most northern areas and highest elevations. See Figure 20 and Figure 21.


 


**Remarks.** Needham [18] described *A. ludens* from larvae and female subimagos collected in the Hasenclever Hills of central New York. Much subsequent effort to obtain male imagos by Needham and others, including Morgan [19] and Clemens [21], was unsuccessful (although Clemens found one presumed male subimago which failed to transform). Morgan [19] was the first to suggest parthenogenesis in *A. ludens*, and Clemens [21] demonstrated parthenogenetic hatching of eggs. In 1924, Needham [20] described what he believed to be the male of *A. ludens*, from a single imago captured in flight. The basis for its association with *A. ludens* was the fact that no other *Ameletus* species was known from the eastern Nearctic at the time. Genitalia from that specimen were illustrated by Traver in Needham et al. [13] but have since been lost. Although Traver’s illustration lacks sufficient detail for the penes, the distinctive dilation in the long joint of the forceps makes it likely that the specimen was *A. patriludens*.

The imagos of both *A. ludens* and *A. lineatus* have ganglionic markings on the venter of the abdomen that vary from barely perceptible to rather distinct on abdominal segments 2 or 3 to 8, and contrary to indications by Traver [13,16] and Burks [17], the two species cannot be separated by their presence or absence (see Remarks under *A. immaculatus*). Nor is the shape of the distal margin of sternum 9 consistently different, as illustrated by Figures 238 and 239 in Burks [17]. And contrary to Traver’s ([13]: 448) key, there is no “white line present between each lateral stripe and pleural fold” in *A. lineatus* larvae. In both *A. ludens* and *A. lineatus*, the lateral edge of each lateral stripe borders the pleural fold. The difference between the two species is most evident in the median stripe (see description above and Figure 16E). However, the ventral stripes on larvae do not fully develop until the last one or two instars (see Figure 16C). At least partly due to this limitation, identifications in the literature are usually unreliable. The best way to assess larval developmental stage is by the extent of the developing wing pads: if the distal tip of the forewing pad does not reach at least as far as the posterior margin of tergite 2, the ventral stripes are likely not fully developed.


 
***Ameletus matrilineatus* sp. nov.**
(Figure 9A–F, Figure 15F and Figure 16C,D) 


**Description.** Male imago (Figure 9A) body length 14 mm, forewing length 12.5 mm. In life, head blackish with opaque white patches on face and between compound eyes and antennae; compound eyes greenish yellow dorsally and gray ventrally with dark line dividing in living individuals. Prothoracic tergum blackish; mesotergum yellowish and brown, with yellow streaks along anterolateral margin, scutellum yellow changing to dark brown and black at the posterior lateral areas; metatergum blackish; thoracic pleuron brown with pale at the base of wings and mesothoracic spiracle. Fore legs brown; middle and hind legs light brown. Wings transparent with brown longitudinal veins and cross-veins. Abdominal tergites with conspicuous tracheae; tergite 1 dark brown; tergites 2–9 yellowish with dark brown shading on posterior margin that extends and forms triangular posterior lateral patches; tergite 10 light brown with dark brown submedian streaks. Abdominal sternites with ganglionic markings on sternites 2–7; sternite 1 brown; sternites 2–8 largely pale yellowish, with conspicuous tracheae; sternite 9 and styliger plate yellow brown with brown spots on styliger plate near the base of each forcep. Long joint of forceps distinctly swollen in distal 2/3 (Figure 9B). Penes (Figure 9C) with ventral plates forming a prominent spine with a row of 4–7 teeth on the ventral margin (Figure 9D). Caudal filaments brown with a narrow, darker ring at the base of each segment. Female imago (Figure 9E) indistinguishable from most other *Ameletus* females. However, female *A. matrilineatus* imagos can be distinguished from those of *A. burkei* or *A immaculatus*, with which it may be found, by the presence of distinct ganglionic markings on the sternites. Larva: identical to *A. lineatus* (see Figure 7D and Figure 16C–D) except for sexuality and ploidy level.


 


**Type Material.** Reared male holotype: Specimen AA_SSC1_265, Station Spring Creek, Burkes Garden, Tazewell County, Virginia, USA (37.095416° N, 81.383856° W, elev. 972 m) 15 April 2024, D.H.Funk and A.C.Graham. Allotype female and paratype male and female, same data, 13 May 1990, D.H.Funk. Types will be deposited at PERC.


 


**Etymology.** *matri* = mother (Latin), named for giving rise to the parthenogenetic *A. lineatus*.


 


**Diagnosis.** *Ameletus matrilineatus* is a diploid bisexual species. Male imagos can be distinguished from all other eastern Nearctic species except *A. patriludens* by the following combination of characters: penes with long narrow lateral lobes extending beyond the base of the forceps; ventral plates forming a prominent spine with a row of about 6 teeth on ventral margin; long joint of forceps with distinct dilation in apical 2/3. *Ameletus matrilineatus* and *A. patriludens* males are very similar, but the pointed extension formed by the ventral plate is longer in *A. matrilineatus* (Figure 15F) and the dilation on the long joint of the forceps is more pronounced in *A. patriludens* (compare Figure 9B with Figure 11B). COI sequences from the two differ by about 6%. Larvae of *A. matrilineatus* are distinguished from other eastern Nearctic species by the same combination of characters used to distinguish *A. lineatus* (see diagnosis under that species). From *A. lineatus, A. matrilineatus* be distinguished only by ploidy level and the presence of males.


 


**Distribution.** Presently known only from southwestern Virginia.


 


**Phenology.** *Ameletus matrilineatus* is univoltine, with an obligate summer egg diapause. Young larvae first appear in autumn and grow through winter and early spring. Adults first appear in mid-April and emergence extends through May.


 


**Remarks.** *Ameletus matrilineatus* is the bisexual progenitor of the triploid parthenogen *A. lineatus*, and the maternal source of the hybrid triploid parthenogen *A. ludens*. *Ameletus matrilineatus* probably once had a broader geographic distribution but is now quite rare and local, possibly having been displaced by its parthenogenetic offshoots. The type locality at Station Spring Creek is just upstream from where that stream sinks into the limestone floor of the Appalachian cove known as Burke’s Garden. The upper reaches of Station Spring Creek have no surface connection with other streams or rivers, and are devoid of fish. (The latter was confirmed in 2024 using an eDNA kit from Jonah Ventures, Boulder Colorado.) *Ameletus matrilineatus* is abundant at that site, but neither of the parthenogens that arose from *A. matrilineatus* (*A. lineatus* and *A. ludens*) are present. In 1990 I collected and reared 261 specimens on 4 April (prior to the start of emergence) and the sex ratio was 53% male, 47% female. I incubated unfertilized eggs dissected from 25 of those females with the following results: 16 clutches with zero hatch, 3 clutches each yielded a single hatchling, for 2 clutches two hatchlings resulted, 1 clutch each at three and five hatchlings, and 2 clutches with nine hatchlings. Each of these clutches contained >1000 eggs, which means parthenogenetic hatch rates were all less than 1%. Parthenogenetic hatch rates for *A. ludens* and *A. lineatus* are >90%. A collection of 85 individuals on 15 April 2024 (emergence period was already underway) yielded 48% male and 52% female, indicating no change over the intervening 34 years.

There may be other extant populations of *A. matrilineatus* yet to be discovered. In the Virginia Tech collection there is a single male reared from Mill Creek, Montgomery Co., VA (37.261448° N, 80.340619° W), 12 April 1979 by Smith.


 
***Ameletus ohioensis* sp. nov.**
(Figure 10A–G and Figure 15B) 


**Description.** Male holotype imago (fresh, in alcohol) (Figure 10A).

Body length 8.8 mm, forewing length 8.7 mm. Head blackish-brown with paler patches on face and between compound eyes and antennae and on vertex; compound eyes pale yellow-green dorsally with a black line laterally, light gray immediately below, then dark gray below that. Prothoracic tergum dark brown; mesotergum light reddish-brown, scutellum light reddish-brown changing to blackish at the posterior lateral areas; metatergum dark brown; thoracic pleuron dark reddish-brown with paler areas at the base of wings, legs and surrounding mesothoracic spiracle. Fore legs light brown; mid- and hind legs pale creamy. Wings transparent. Longitudinal veins amber brown, cross-veins light brown. Abdominal tergite 1 dark brown; tergites 2–9 yellowish with brown shading on posterior margin that extends and forms triangular posterior lateral patches; tergite 10 yellowish with brown submedian streaks. Abdominal sternite 1 blackish; sternites 2–8 creamy yellowish with brown submedian spots and faint ganglionic spots; 9 yellowish. Styliger plate creamy yellowish; forceps gray-brown. Caudal filaments light brown with a reddish-brown ring near the base of each segment. Long joint of forceps with uniform thickness through its length (Figure 10B). Penes (Figure 10C and Figure 15B) with ventral plates not produced into a spine posteriorly, with a row of 3–7 teeth along the ventral margin (Figure 10D)

Larva: Body length 8–10 mm (holotype 9.3 mm). Dorsal color pattern subtle and variable. Sternal coloration also variable (Figure 10F–G), but usually pale creamy with conspicuous brown maculae on median and on lateral margin (Figure 10F). Mandible with distinct setal gap, 20–40% length of setal row (Figure 10E). Legs not distinctly patterned except for apical black band on tarsi and sometimes another band at tarsal base. Small spines always present on posterior margins of terga 4–10; most individuals also have smaller spines on tergum 2 and 3, which may be scattered. Gill 4 of mature larvae with anal rib narrowly inset, with spines present on both anterior and posterior margins (as in Figure 5E). Caudal filaments pale at base with a dark band in middle 1/3 (with all articulations similarly colored) and with distal tips darkened.


 


**Type Material.** Male imago holotype. Reared from larva, tributary of Storms Creek, 1.6 km E of Ellisonville, Lawrence County, Ohio, USA (38.606389° N, 82.629167° W, elev. 181 m), 7 May 1987, D.H.Funk. Two male imago paratypes, reared from larvae, same data. Types will be deposited at PERC.


 


**Etymology.** named for its known distribution, in small tributaries along the Ohio River in Ohio and West Virginia.


 


**Diagnosis.** *Ameletus ohioensis* is a diploid bisexual species. Adult males can be distinguished from all other eastern Nearctic *Ameletus* by the following combination of characters: long lateral lobes of the penes extending beyond the base of the forceps; long joint of forceps with uniform thickness (i.e., without distinct dilation in apical 1/3); ventral plates of the penes neither produced into a spine distally, nor with a prominent rounded lobe projecting anteriorly, but extending ventrally into a narrow plate with 3–7 teeth along its ventral margin (Figure 10D and Figure 15B). Larvae can be distinguished from most other eastern Nearctic species by the following combination: distinct but narrow setal gap on the mandible; gill 4 with spines on both the costal and anal margins, with anal rib narrowly inset (as in Figure 5E); dark band in middle 1/3 of caudal filaments with segments uniformly colored; venter of abdomen usually with distinctive maculation (Figure 10F) but this may be less developed in some specimens (Figure 10G). Larvae with paler venter and older, faded specimens are not distinguishable from *A. immaculatus* other than by the presence of males, or from *A. tarteri* or *A. ohioensis* other than known geographic range or COI haplotype.


 


**Distribution.** Southeastern Ohio and adjacent West Virginia.


 


**Phenology.** *Ameletus ohioensis* is probably univoltine with an obligate summer egg diapause, but this has not been determined experimentally. Emergence is largely in the month of May.


 


**Remarks.** I have collected *A. ohioensis* from several localities in Lawrence and Washington Counties, OH as well as Cabell County, WV. I have examined specimens (originally reported as male *Ameletus ludens*) from Vinton and Guernsey Counties, OH, by Hall [22]. I have collected both *A. lineatus* and *A. immaculatus* together with *A. ohioensis* in Ohio and West Virginia.


 
***Ameletus patriludens* sp. nov.**
(Figure 11A–G and Figure 15E) 


**Description.** Male holotype imago (fresh, in alcohol) (Figure 11A). Body length 10.5 mm, forewing length 10.5 mm. In life, head blackish with paler patches on face and between compound eyes and antennae; compound eyes greenish yellow dorsally and dark brown ventrally with dark line dividing in living individuals. Prothoracic tergum blackish; mesotergum yellowish and brown, with yellow streaks along anterolateral margin, scutellum light brown changing to black at the posterior lateral areas; metatergum blackish; thoracic pleuron dark brown with paler areas at the base of wings and mesothoracic spiracle. Fore legs brown; middle and hind legs light brown. Wings nearly transparent with a slight brownish stain. Longitudinal veins and cross-veins dark brown. Abdominal tergites with conspicuous tracheae; tergites 2–9 yellowish with a light brown area on the median and dark brown shading on posterior margin that extends and forms triangular posterior lateral patches; tergite 10 light brown with dark brown submedian streaks. Abdominal sternites with ganglionic markings on sternites 2–7; sternite 1 brown; sternites 2–8 largely pale creamy, with conspicuous tracheae; sternite 9 light brown with dark brown on anterior and lateral margins and styliger plate light brown with darkened distolateral points and brown spots near the base of each forcep. Long joint of the forceps distinctly dilated in distal 2/3 (Figure 11B). Penes (Figure 11C) with ventral plates forming a prominent spine with a row of 3–5 teeth on the ventral margin (Figure 11D). Caudal filaments brown with darker rings at joints. Larva: indistinguishable from *A. ludens* except for ploidy level, presence of males and COI haplotype.


 


**Type Material.** Reared male holotype: Specimen AL_Nev_57, West Branch Neversink River at Slide Mtn W Trailhead, Sullivan County, New York, USA (42.00866° N, 74.427118° W, elev. 613 m) 21 April 2022, D.H.Funk. Allotype female (AL_Nev_101) and paratype male (AL_Nev_71), same data. Types will be deposited at PERC.


 


**Etymology.** *patri* = father (Latin), paternal source of the hybrid triploid parthenogenetic *A. ludens*.


 


**Diagnosis.** *Ameletus patriludens* is a diploid bisexual species. Male imagos can be distinguished from all other eastern Nearctic species except *A. matrilineatus* by the following combination of characters: penes with long narrow lateral lobes extending beyond the base of the forceps; ventral plates forming a prominent spine with a row of about six teeth on ventral margin; long joint of forceps with distinct dilation in apical 2/3. *Ameletus patriludwns* and *A. matrilineatus* males are very similar, but the pointed extension formed by the ventral plate is shorter in *A. patriludens* (Figure 15E) and the dilation of the forceps long joint is slightly greater in *A. patriludens* (compare Figure 11B and Figure 9B). COI sequences from the two differ by about 6%. Larvae of *A. patriludens* are distinguished from other eastern Nearctic species by the same combination of characters used to distinguish *A. ludens* (see diagnosis under that species). The ventral stripes in *A. patriludens* are quite similar to those of *A. ludens:* the median stripe is broader and its margins are less defined compared to those of *A. lineatus* and *A. matrilineatus* (Figure 16E), and the same is true for the lateral stripes to a lesser degree. However, in *A. patriludens*, the stripes often do not coalesce on sternite 9 to the degree they do in *A. ludens*, such that white patches between them are evident over most of that segment (Figure 11F), and in some cases, the stripes do not coalesce at all (Figure 11G). However, *A. patriludens* larvae can only be distinguished with certainty from *A. ludens* by ploidy level, the presence of males or COI haplotype.


 


**Distribution.** Presently known only from two localities: the type locality in New York and another in north-central Pennsylvania (see Remarks below).


 


**Phenology.** *Ameletus patriludens* is univoltine, with an obligate summer egg diapause. Young larvae first appear in autumn and grow through winter and early spring. Adults first appear in mid-May, and emergence extends into early June.


 


**Remarks.** *Ameletus patriludens* is the paternal progenitor of the hybrid triploid parthenogen *A. ludens*, whose maternal source is *A. matrilineatus*. Like its bisexual relative *A. matrilineatus*, *A. patriludens* probably once had a broader geographic distribution but is now rare and local, possibly having been displaced by its parthenogenetic offshoots. At the type locality on the upper West Branch of the Neversink, neither *A. lineatus* nor *A. ludens* are present. On 21 April 2022 I collected 106 larvae and found a sex ratio of 58% male and 42% female. Thirty-five larvae were sequenced for COI, with the remaining tissue frozen for flow cytometric determination of ploidy. Sixty-two larvae were reared to adults. Of the 45 individuals sequenced, 43 had the same haplotype, while 2 (female larvae) had a second haplotype that differed by a single base substitution (A for G at position 474). Unfertilized eggs were dissected from seven females with the common haplotype, and the parthenogenetic hatch rates for those ranged from <1 to 22%.

In March of 2024, David I. Rebuck and I discovered a population of *A. patriludens* in an unnamed tributary of Elk Creek at Gramley Gap, Center Co. Pennsylvania (40.94156° N, 77.40467° W, elev 418 m). These have the same haplotype as predominates at the Neversink. There may be other extant populations of *A. patriludens* yet to be discovered.


 
***Ameletus tarteri* Burrows**
(Figure 12A–F and Figure 15G) 


*Ameletus tarteri* Burrows [23]: 284; Zloty [1]: 315.


 


**Type Material.** Male imago holotype, reared with exuviae. Hamrick Run at West Virginia Route 39/55 near confluence with North Fork of Cherry River, Greenbrier County, West Virginia, USA (38.22778° N, 80.40111° W, elev. 900 m), 15 June 1983, W. L. Burrows. Deposited at USNM (not examined). Male and female paratypes from the type locality and adjacent Carpenter Run, deposited at USNM, CNC, MU, PERC, FAMU, and ANSP.


 


**Diagnosis.** *Ameletus tarteri* is a diploid bisexual species. Male imagos (Figure 12A) can be distinguished from other eastern Nearctic *Ameletus* species by the following combination of characters: long lateral lobes of the penes extending beyond the base of the forceps; long joint of forceps with uniform thickness (i.e., without dilation in apical 1/3; Figure 12B); ventral plates of the penes produced into a prominent spine distally (Figure 12C and Figure 15G), with 1–4 teeth along its ventral margin (Figure 12D).

Larvae can be distinguished from most other eastern Nearctic species by the following combination: distinct but narrow setal gap on the mandible (Figure 12F); gill 4 with spines on both the costal and anal margins, with anal rib narrowly inset (as in Figure 5E); dark band in middle 1/3 of caudal filaments with segments uniformly colored; venter of abdomen without stripes or distinctive maculation. Not distinguishable from *A. immaculatus* other than by the presence of males or COI haplotype, or from *A. burkei* other than known geographic range or COI haplotype.


 


**Distribution.** Eastern West Virginia and western Maryland.


 


**Phenology.** In contrast to its closest relatives, larvae of *A. tarteri* are present throughout the year. Matthews and Tarter [24] reported a univoltine life cycle with a bimodal pattern of emergence, one period occurring in late May to early July and a second period in September. I have found a lack of summer egg diapause and an extended period of hatching in *A. tarteri*, suggesting larval recruitment is more or less continuous throughout the year.


 


**Remarks.** Burrows [23] described *A. tarteri* from a small area in SE West Virginia. Both the type locality, Hamrick Run, and the adjacent Carpenter Run, where Matthews and Tarter [24] studied its life history, had very low pH in the 1980s (~4.5). In 1990, I surveyed both streams and found *A. tarteri* to be the only *Ameletus* species present. However, at several nearby sites with a more circumneutral pH (in the 6.5–7.75 range), I found *A. tarteri* together with *A. immaculatus* and *A. rebucki*. In 2022, I encountered a similar situation in east-central West Virginia in tributaries of the Blackwater and Cheat Rivers: *A. tarteri* was the only *Ameletus* present in Shay’s Run and Red Run, where pH was 4.4, but at several nearby circumneutral sites (pH 6.5–8.0), *A. tarteri* was accompanied by *A. immaculatus*, *A. rebucki*, *A. ludens*, and *A. lineatus*. Thus, it appears *A. tarteri* is able to tolerate a lower pH than other eastern Nearctic *Ameletus* species. From 1987 to 1990, there was an active coal mine in the upper Hamrick Run (type locality) watershed, followed by a reclamation effort, which has resulted in a sharp increase in water pH; in September of 2022, I measured 8.2 at the type locality. It will be interesting to see if and when other *Ameletus* species colonize that creek.

Burrows [23] reported larval *A. tarteri* (of unspecified gender) from Virginia and New York. However, *A. tarteri* larvae cannot be distinguished with certainty from those of *A.burkei*, *A. ohioensis*, or *A. immaculatus*, if they are female. Given what we now know of the distribution of these four species, Burrows’ Virginia and New York specimens were almost certainly *A. immaculatus*.


 
***Ameletus subnotatus* species group**
 


Three species from the Nearctic, the transcontinental *A. subnotatus*, the Western *A. oregonensis* and the Eastern *A. walleyi*, form a distinct group diagnosed by the unique shape of the male genitalia [1], with short, broad lateral lobes not reaching to the base of the forceps (Figure 2 in Zloty [10]; Figure 13D). Larvae of *A. walleyi* are unknown, but together *A. subnotatus* and *A. oregonensis* can be distinguished from other western Nearctic *Ameletus* by the combination of numerous spines on the posterior margins of sternites 6–8 and caudal filaments with alternating dark and light segments (i.e., speckled) as in Figure 13H. These two also have the anal rib of gill 4 inset far from the anal margin (Figure 13F). It is likely that the larvae of *A. walleyi*, once discovered, will share these characters.


 
***Ameletus subnotatus* Eaton**
(Figure 13A–D,F–H) 


*Ameletus subnotatus* Eaton [25]:211; Spieth [26]:92; Zloty [1]: 314.


 


**Type Material.** Male lectotype as designated by Spieth [26]: Colorado, USA; NHM (not examined).


 


**Diagnosis.** *Ameletus subnotatus* is a bisexual species. Male imagos have wings that are mostly transparent with distinctive black patches and margining around some cross-veins, giving them a speckled appearance (Figure 13A). Body length ranges from 7–10 mm, forewing length 8–10 mm. Upper portion of the compound eye dark brown to black in life. Males can be distinguished from most other eastern Nearctic species by the distinctively shaped penes (Figure 13C), the lateral lobes of which do not extend as far as the base of the forceps (Figure 13D vs. Figure 13E). The only other *Ameletus* in the eastern Nearctic with this type of penes is *A. walleyi*, which does not have speckled wings and differs in the fine details of the penes.

Larvae can be distinguished from all other known eastern Nearctic species by the following combination: wide setal gap on the mandible that is about as long as the setal row (Figure 13G); gill 4 with anal rib inset far from anal margin and with spines on both the costal and anal margins (Figure 13F); caudal filaments dark from base to about ¾ of their length, with every fourth segment pale, giving them a speckled appearance (Figure 13H); spines present on posterior margins of tergites 2– or 3–10.


 


**Distribution.** Transcontinental species with known eastern populations restricted to southern Canada (Ontario, Quebec, New Brunswick, and Newfoundland; Zloty 1996) and adjacent areas in the U.S.


 


**Phenology.** I followed this species in rivers near Sept-Iles, Quebec, where emergence occurred in mid- to late-June and young larvae reached collectable size by early September. There, *A. subnotatus* appears to be univoltine, without a summer egg diapause, but this has not been thoroughly documented.


 


**Remarks.** *Ameletus subnotatus*, *A. walleyi*, and the western North American *A. oregonensis* form a distinct group based on male genitalia. Larval specimens from Quebec have caudal filament coloration as described above and shown in Figure 13H. However, Zloty’s [10] description based on material from Alberta differs: “Caudal filaments with a few brown basal segments followed by a light brown band (about one-third of each filament’s length), followed by a band consisting of alternating brown and pale rings (covering about one-third of each filament’s length), followed by a pale band, and a few brown apical segments.” The taxonomic significance, if any, of this difference is unclear.


 
***Ameletus walleyi* Harper**
 


*Ameletus walleyi* Harper [27]: 603; Zloty [1]: 319.


 


**Type Material.** Male holotype and female allotype: Headwaters of the Eramosa River, Wellington County, Ontario, Canada, 3 and 1 May 1969, respectively, P.P. Harper; in CNC (one male paratype examined)


 


**Diagnosis.** *Ameletus walleyi* is a bisexual species. Male imagos have wings that are mostly transparent with amber shading at the base and with dark brown longitudinal veins and brown cross-veins (Figure 44A in Zloty [1]). Body length ranges from 11 to 12 mm, forewing length 10 mm. Upper portion of the compound eye dark brown to black in life. Males may be distinguished from most other eastern Nearctic species by the distinctively shaped penes (similar to Figure 13C), the lateral lobes of which do not extend as far as the base of the forceps (Figure 13D vs. Figure 13E). The only other *Ameletus* in the eastern Nearctic with this type of penes is *A. subnotatus*, which has distinctively speckled wings and differs in the fine details of the penes (Figures 35 and 36B in Zloty [1]).

Larvae are unknown.


 


**Distribution.** Known only from two localities in southern Ontario (Wellington County).


 


**Phenology.** The only known specimens were collected as adults around the first of May.


 


**Remarks.** *Ameletus walleyi*, *A. subnotatus*, and the western North American *A. oregonensis* form a distinct group based on male genitalia. Larvae of *A. walleyi* are currently unknown, but are likely similar to *A. subnotatus*.


 
***Ameletus tertius* species group**
 


Among the eastern Nearctic *Ameletus*, male imagos of this group can be distinguished by the basal lobes of the ventral plates of the penes (Figure 14C) and the relatively long accessory teeth on the ventral margin of those plates. Larvae lack spines on the anal margin of gill 4 and the anal rib is not inset (Figure 14H). Here, I consider this group monotypic, but two COI haplotype groups from the southern Appalachians, which differ considerably from both the northern group and each other, may eventually prove worthy of specific status (see Remarks below and Section 4.1.1).


 
***Ameletus tertius* McDunnough**
(Figure 14A–I and Figure 15A) 


*Ameletus tertius* McDunnough [28]:27; Zloty [1]: 316.


 


**Type Material.** Male holotype and female allotype: Baddeck Forks, Cape Breton Island, Nova Scotia, Canada 13 July 1936, J. McDunnough; No. 4287 in CNC (male paratype examined).


 


**Diagnosis.** *Ameletus tertius* is a diploid bisexual species. Male imagos (Figure 14A) are clear-winged with a distinct amber tinge in the costal region of the forewing, with light brown veins and cross-veins. Upper portion of the eyes light green in life. They can be distinguished from other eastern Nearctic species by the following combination of characters: penes with long lateral lobes extending beyond the base of the forceps; long joint of forceps with uniform thickness through its length (Figure 14B); ventral plates of the penes narrow, not produced into a spine distally (Figure 15A), with prominent rounded basal lobe projecting anteriorly (Figure 14C) and 4–7 relatively long (18–23 µm) spinules along ventral margin (Figure 14D).

Larvae can be distinguished from all other eastern Nearctic species by the following combination: no setal gap on the mandible (Figure 14I); gill 4 with spines on the costal but not anal margins, with anal rib on anal margin (i.e., not inset; Figure 14H); dark band in middle portion of caudal filaments evenly colored (Figure 14E) and may extend to the base (Figure 14F); dorsum of abdomen distinctively patterned, with tergites 2–6 gray-brown with large submedian pale spots, tergites 7–8 lighter brown and 9–10 dark (Figure 14E); venter without dark stripes running the length of the abdomen but sternite 8 with median and sublateral dark maculae and sternite 9 almost entirely dark (Figure 14G); spinules present on posterior margins of all tergites.


 


**Distribution.** Quebec and Labrador, the Canadian Maritimes, and New England south to North Carolina.


 


**Phenology.** Univoltine, with emergence in spring to mid-summer (generally later than other eastern Nearctic species). May have a summer egg diapause.


 


**Remarks.** Although the penes of *A. tertius* resemble those of *A. burkei* and *A. ohioensis*, larval characters and genetic data (both COI sequences and allozymes) indicate *A. tertius* is not closely related to any other eastern Nearctic species. Although populations from Pennsylvania northward are genetically uniform (as expected due to the recency of their post-glacial dispersal into this area from refugia in Pennsylvania), there is considerable differentiation in mitochondrial COI between these and populations from the southern Appalachians (see Section 4.1.1). Larval coloration in some North Carolina populations is distinctive in that the dark band on the caudal filaments extends from, or nearly from, their base (Figure 14F). The latter are treated as “*A.*sp.3” in the larval key of Morse et al. [29], but adult males I have reared from these have genitalia identical to those from larvae having the more typical color pattern.

## 4. Discussion

### 4.1. Justification for Taxonomic Decisions

I recognize four species groups among *Ameletus* in the eastern Nearctic (south of the Arctic zone), all of which appear to be monophyletic (see justifications below): the *browni* group, including *A. browni*, *A. cryptostimulus*, and *A. rebucki*; the *ludens* group, including *A. burkei*, *A. immaculatus*, *A. janetae*, *A. lineatus*, *A. ludens*, *A. matrilineatus*, *A. ohioensis*, *A. patriludens*, and *A. tarteri*; the *A. subnotatus* group, which includes the transcontinental *A. subnotatus*, the western Nearctic *A. oregonensis* and the Eastern *A. walleyi*; and the *tertius* group, which may actually represent a species complex but which at this time I conservatively consider monotypic.

Grouping of species was based on morphology and phylogenetic analysis of COI, which was sequenced for 450 of the specimens collected during this study. Allozyme data were critical to interpretations within the *A. ludens* group, which includes the three polyploid parthenogenetic species. (Although allozyme data support the taxonomic interpretations presented herein for members of the *A. browni* and *A. tertius* groups, data were only available for a subset of the populations for which COI was sequenced and thus are not presented here.)

**Figure 18:**
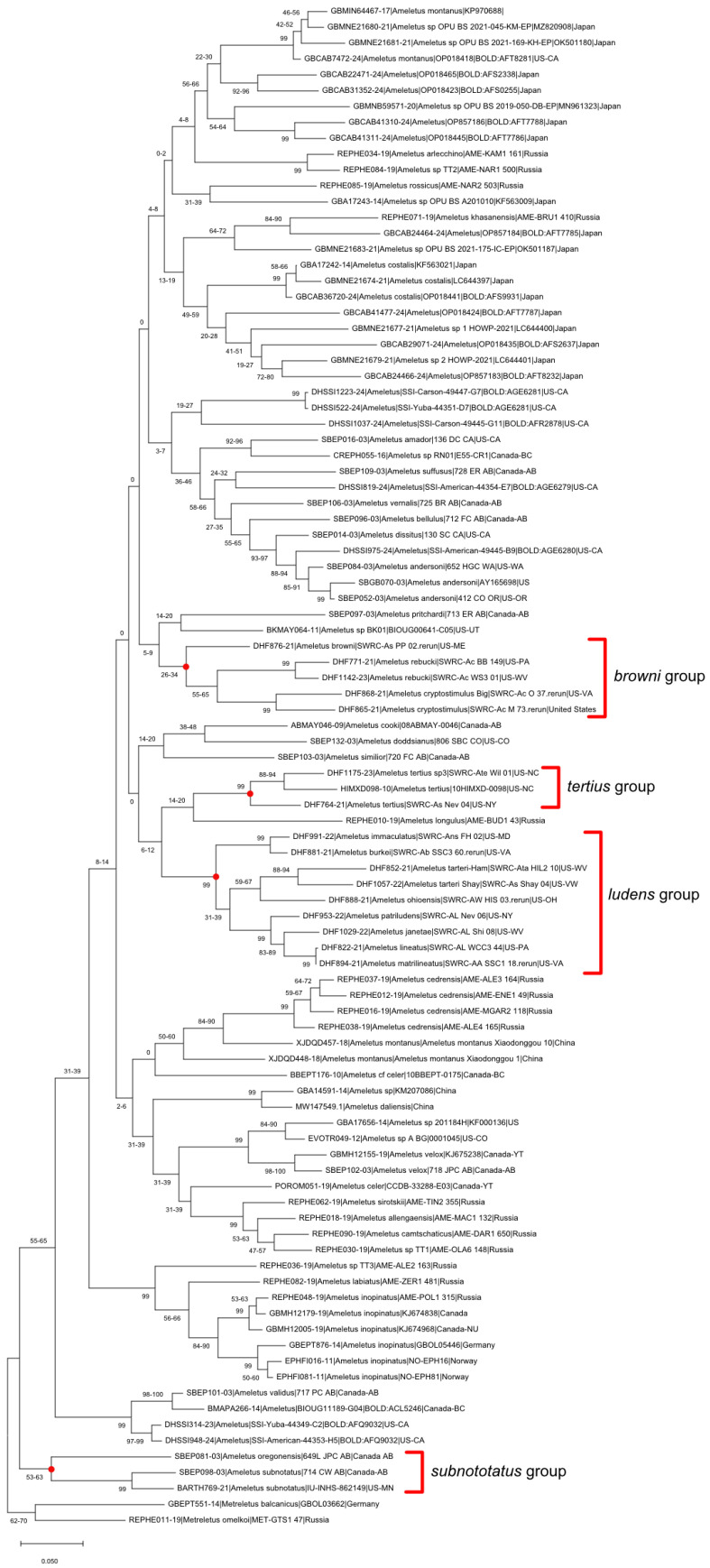
Maximum likelihood tree based on COI sequences from 96 ameletid mayflies representing all 94 BINs in BOLD. Two *Metreletus* species were designated as outgroup. Numbers on branches indicate bootstrap values after 117 replications (determined adaptively). Specimens are identified by sequence ID, taxon, specimen ID, and geographic origin.

In addition to the 450 COI sequences generated during this study, 1178 Ameletidae sequences were available on BOLD (as of January 2025), from which 96 were chosen (representing 94 BINs) for the maximum likelihood tree shown in Figure 18. Together the two *Metreletus* species were designated as outgroup. All four of the above species groups were recovered. Although bootstrap support for the *subnotatus* group and the *browni* group was low (53–63 and 55–65, respectively), these two are well supported morphologically. Bootstrap support was good for the *tertius* group (99) and the *ludens* group (99), and within the latter, the *ludens* complex (see below) was also well supported (99). Deeper nodes in the tree were poorly supported.

**Figure 19:**
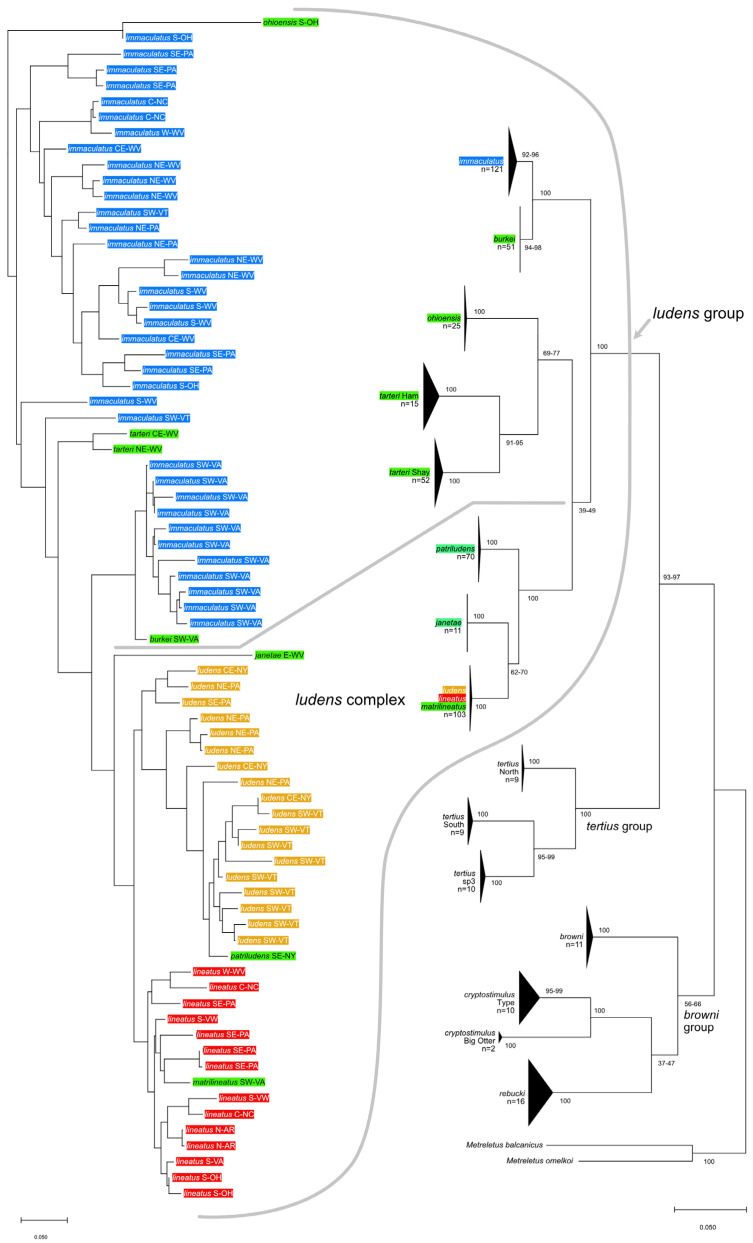
Right side: Maximum likelihood tree generated for 515 COI sequences from the eastern Nearcic *Ameletus* and 2 (Palearctic) *Metreletus* (outgroup). Numbers on branches indicate bootstrap values after 115 replications (determined adaptively). Subtrees were compressed in Mega12. The vertical dimension of the resulting triangles is proportional to the number of individuals included, up to *n* = 12, beyond which that dimension is fixed. Left side: NJ tree constructed from genetic distances (D_A_) based on allozyme data from 23 loci. Bisexual species are colored green (see Methods for n values). Parthenogenetic clones are identified by species and geographic origin (region-US state) and are color-coded as follows: *immaculatus* blue; *ludens* orange; *lineatus* red.

The COI tree on the right side of Figure 19 includes sequences from 515 *Ameletus* from the eastern Nearctic and two *Metreletus* species designated as outgroup. Three of the species groups I recognize are represented here (we have no sequences for *A. walleyi* and no sequences for *A. subnotatus* from the eastern end of its range, so the *A. subnotatus* group is not represented).

#### 4.1.1. The *A. tertius* Species Group

The *A. tertius* group in Figure 19 includes three distinct branches. *Ameletus tertius* was originally described from Nova Scotia and is found throughout the Northeast, from Quebec and New Brunswick south to Pennsylvania. It is also known from scattered localities in western North Carolina and adjacent Georgia and Tennessee. COI has been sequenced for 28 *A. tertius* from New York, Pennsylvania, and North Carolina. The three haplotype clusters in Figure 19 differ from each other by 7.5–9.3%. One is from the Northeast (Pennsylvania and New York: labeled *tertius* North), and two are from the southern Appalachians (labeled *tertius* South and *tertius* sp3). Genitalia from adult male representatives of the three haplotype groups appear identical. The only apparent morphological difference among these groups is in the coloration of the larval caudal filaments: for individuals from the group labeled *tertius* sp3 the dark band on the caudal filaments extends from their base (or near their base) to about 2/3 their length (Figure 14F); in larvae from the other two haplotype groups the caudal filaments are pale in the basal 1/4 (Figure 14E). These haplotype groups might indicate the presence of cryptic species, but in the absence of corroborative evidence, such as nuclear markers or discreet morphological differences, I conservatively consider these to represent a single species, *A. tertius*.

#### 4.1.2. The *A. browni* Species Group

The 39 COI sequences from the *A. browni* group mostly sort in accordance with the species concepts presented in Section 3.4. *Ameletus browni* ranges from Quebec south to Pennsylvania, and all known populations are within the region covered by ice during the late Wisconsin glaciation of the Pleistocene. Not surprisingly, COI shows little variation. *Ameletus rebucki* ranges from northern New England south and west to eastern West Virginia. COI is quite uniform within the recently glaciated area, but is more variable among the West Virginia populations. *Ameletus cryptostimulus* is known from the mountains of central Virginia to South Carolina and eastern Tennessee. COI haplotypes are similar among samples from the type locality in southwest Virginia and populations from North Carolina and eastern Tennessee, but the northernmost population sampled, from the headwaters of the Big Otter River in Virginia (labeled as *cryptostimulus* Big Otter in Figure 19), differs by about 9.9% (K2P distance) from the others. Morphologically, this population is indistinguishable from those at the type locality in Virginia and samples from North and South Carolina. As with the *A. tertius* clusters, with no evidence other than COI to support splitting the *A. cryptostimulus* clusters, I conservatively treat them as conspecific.

#### 4.1.3. The *A. ludens* Species Group: *ludens* Complex

Among the species included in the *A. ludens* group, the following constitute monophyletic subgroup which I will refer to as the *ludens* complex: *A. lineatus*, *A. ludens*, *A. janetae*, *A. matrilineatus*, and *A. patriludens*. The *ludens* complex can be distinguished by a slight to prominent dilation in the apical third of the long joint of the forceps in the adult male, and in the larvae, distinct dark stripes on the venter of the abdomen and pale speckling within the dark band of the caudal filaments.

Together, the two obligately parthenogenetic species in this complex, *A. ludens* and *A. lineatus*, have by far the broadest geographic range and account for the vast majority of all *Ameletus* encountered in eastern North America. Morphologically, these two can only be distinguished by minor differences in the ventral stripes of larvae. However, our ability to identify specimens this way is complicated by the fact that the shape and extent of the ventral stripes changes considerably during larval development (see Figure 16), and as a result, the species can only be distinguished in the final larval instars (see Remarks under *A. ludens* in Section 3.4).

**Figure 20:**
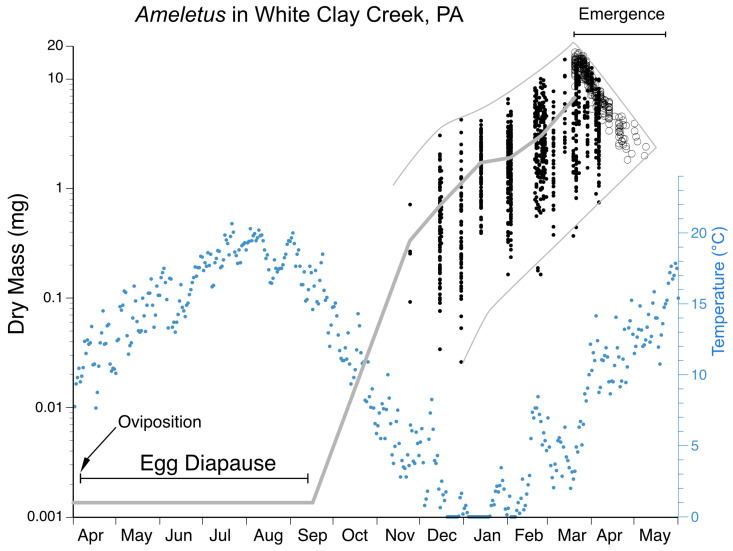
Life history of *Ameletus ludens* and *lineatus* (combined) in White Clay Creek, Pennsylvania, USA. Thick gray line represents mean dry mass. Thin gray line encloses dry mass distribution from late autumn until the end of emergence in spring. Black dots represent individual larvae, and open circles represent emerging females. Mean daily water temperature is superimposed in blue.

**Figure 21:**
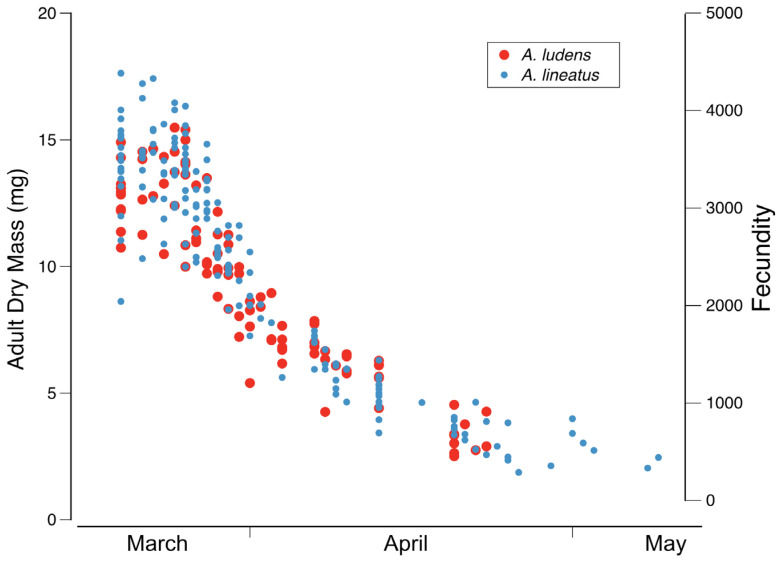
Adult dry mass and fecundity over the emergence period for *Ameletus ludens* and *lineatus* in White Clay Creek, Pennsylvania, USA.

Both *A. ludens* and *A. lineatus* are common in White Clay Creek, southeastern Pennsylvania, USA, where I, together with colleagues Robin L. Vannote, Bernard W. Sweeney, Jay W. Richardson and others, have documented their life history and genetics. Regular field collections throughout the year, supplemented by incubation of eggs in the laboratory, enabled us to determine the pattern illustrated in Figure 20. We found that emergence (and oviposition) begins in late March and extends through April and into early May. Eggs spend the warm summer months in diapause, and hatching does not begin until late September when water temperature drops to about 15 °C. Hatchlings are ca. 1 µg dry mass, and larvae grow rapidly in autumn but do not reach a field-collectable size until early November. Growth rate slows somewhat in January and early February, then increases through the later part of winter and early spring prior to adult emergence. *Ameletus ludens* and *A. lineatus* appear to have identical life histories in White Clay Creek, but because the two can only be distinguished morphologically in the final larval instars, the larval weights shown in Figure 20 represent a combination of the two. To determine adult dry mass, we brought those field-collected larvae with black wingpads (an indicator that emergence will occur in the next day or two) into the laboratory and reared them at ambient White Clay Creek temperature. The emerging adults could then be identified by the stripes on the associated larval exuviae. Figure 21 shows the size distribution of those individuals over the emergence period. The two species follow a virtually identical pattern in both size and timing of emergence, with adult size and fecundity dropping off dramatically in both as the emergence period progresses. At the beginning of emergence adults weigh ca. 16 mg and contain ca. 4000 eggs, but by the end of the emergence period adults are closer to 2 mg with only a few hundred eggs.

Because *Ameletus ludens* and *A. lineatus* in White Clay Creek were indistinguishable in every respect other than larval color pattern, I initially suspected that, rather than signifying distinct species, these color patterns might instead reflect either a genetically based polymorphism or ecophenotypes. However, allozyme data revealed the two have distinct multi-locus genotypes (MLGs) (Figure 15H). And rearing experiments showed them to reproduce clonally (all offspring being identical to their mothers) with both the *A. ludens* and *A. lineatus* color patterns breeding true.

Comparisons of allele frequencies between sympatric sexually reproducing species usually reveal one or more fixed differences (i.e., loci at which the two share no alleles; e.g., Funk et al. [30]). If these *A. ludens* and *A. lineatus* clonal parthenogens each represent a “frozen” genotype inherited from different bisexual parent species, one might expect to see a genetic pattern similar to what we observe in bisexual species, with no shared alleles at some loci. Although the *A. ludens* and *A. lineatus* MLGs illustrated in Figure 15H differ at fully half of the loci scored (14 of 28), they share at least one allele at each of these loci. Thus, the pattern seen in Figure 15H would not rule out their having both arisen from a single bisexual population.

Some of the banding patterns depicted in Figure 15H are quite complex. This is partly due to the subunit structure of the particular enzyme in question (for an example of allelic interpretation of banding patterns for a dimeric locus, see Figure 17F). Nevertheless, banding patterns for both species/clones were strongly suggestive of polyploidy. For example, for the enzyme *Mpi* the *A. ludens* clone stained as three bands and the *A. lineatus* clone only two, one of which was distinctly stronger than the other. *Mpi* is monomeric and thus a diploid individual that is heterozygous is expected to show two bands of equal intensity. The *A. ludens* clone in this case displays three different alleles, and while the *A. lineatus* clone has only two, it appears to have a double dose of the faster allele.

Another feature of these clones is that heterozygosity (measured as the proportion of loci that are heterozygous) is quite high, 0.32 for the *A. ludens* clone and 0.23 for the *A. lineatus* clone in White Clay Creek, compared to an average expected heterozygosity (H_exp_) of 0.09 in bisexual members of the *A. ludens* group (range 0.02 to 0.16; see Table S2). Such a combination of high heterozygosity and polyploidy is strongly suggestive of a hybrid origin.

In light of the above and in order to gain a better understanding of relationships and bisexual origins, my initial survey was broadened geographically to include populations from Maine to North Carolina in the East, and west to Arkansas, and two additional technologies were utilized: the sequencing of a portion of the mitochondrial gene COI (i.e., “genetic barcoding”) to help discern maternal lineages; and flow cytometric determination of genome size in order to document ploidy levels.

Allozymes have revealed considerable clonal diversity (as indicated by the number of allozyme MLGs) for both *A. ludens* (*n* = 29) and *A. lineatus* (*n* = 14). However, barcoding indicates only a tiny amount of variation in COI among these clones. In fact, the *A. ludens* and *A. lineatus* clones from White Clay Creek (illustrated in Figure 15H) have identical COI sequences and that same haplotype has been found in *A. ludens* and *A. lineatus* from 12 states/provinces from Arkansas to Ontario and Maine, south to North Carolina, accounting for 69 of the 94 sequences we now have for those species. Ten additional haplotypes have been found, nine of which differ from the common haplotype by only a single base (0.15%), and the substitutions are transitional rather than translational. A single haplotype from Indiana differs by four bases (0.6%), three of which are translational.

During the course of this survey, two diploid bisexual members of the *A. ludens* complex were discovered, *A. patriludens* sp. nov. in the Catskills Region of New York and *A. matrilineatus* sp. nov. in Burkes Garden in southwestern Virginia (see Section 3.4). The Virginia *A. matrilineatus* population appears to be entirely bisexual: the sex ratio was close to 1:1 (51% male, *n* = 287), and parthenogenetic hatch rates of eggs taken from 25 virgin females were mostly 0% (*n* = 16); the remaining 9 ranged up to 0.4%. Unfertilized hatch rates in parthenogenetic *A. ludens* and *A. lineatus* are ≥90% (Funk, in prep.). Larvae from this *A. matrilineatus* population have ventral stripes identical to those of *A. lineatus*. In a sample of 10 individuals from that population four COI haplotypes were detected, differing from the common *A. ludens/lineatus* haplotype by one (*n* = 5), two (*n* = 2) or four (*n* = 3) bases (a difference of only 0.15%, 0.3% and 0.6%, respectively). (K2P distance among the 4 *A. matrilineatus* haplotypes averaged 0.37%; range 0.15–0.64%.) Thus, it appears *A. matrilineatus* (or something very close to it) is the maternal ancestor of both *A. ludens* and *A. lineatus*.

The New York *A. patriludens* population also appears to be entirely bisexual (56% male, *n* = 106), but parthenogenetic hatch rates, although low, were somewhat higher than in the Virginia *A. matrilineatus* population (range < 1% to 22%; *n* = 7). Of the 56 individuals sequenced from that New York population, 54 had the same COI haplotype. Two female larvae (presumed to be members of the same bisexual population) were found with a haplotype that differed by a single base. Together, these haplotypes differ from the common one found in *A. ludens* and *A. lineatus* by 38 bases (K2P distance = 6.12%).

While the uniformity of mitochondrial COI haplotypes among *A. ludens* and *A. lineatus* suggests a single maternal ancestor, *A. matrilineatus*, a neighbor-joining tree constructed from nuclear (allozyme) data shows the 18 known *A. ludens* clones (red highlight) cluster with *A. patriludens* while the 14 *A. lineatus* clones (orange highlight) cluster with *A. matrilineatus* (Figure 19). Thus, it appears *A. lineatus* clones may have arisen from within an *A. matrilineatus*-like ancestor, but *A. ludens* clones are the result of hybridization between an *A. matrilineatus*-like mother and an *A. patriludens* father. Two other lines of evidence support this conclusion. Heterozygosity is significantly higher in *A. ludens* (mean = 0.277) than in *A. lineatus* (mean = 0.186) (*p* < 0.001 in a *t*-test comparing arcsine square root-transformed heterozygosity values), as expected if *A. ludens* clones are of hybrid origin. And several allelic markers show a pattern consistent with this interpretation. For example, *A. patriludens* is fixed for allele *a* at locus *Aat-1* (Table S3). That allele is absent in *A. matrilineatus* and all known *A. lineatus* clones, but most *A. ludens* are heterozygous for that *A. patriludens* allele and one or two others found in *A. matrilineatus*.

Flow cytometric determination of genome size showed a mean DNA content per nucleus in both *A. ludens* and *A. lineatus* of 1.1 pg (*n* = 14) which is almost exactly 50% greater than that determined for *A. matrilineatus* (0.75 pg; *n* = 3; 1m, 2f) or *A. patriludens* (0.76 pg; *n* = 5; 2m, 3f), as expected if the parthenogens are triploid.

Although none of the four COI haplotypes found in *A. matrilineatus* from the type locality have been found in any of the known *A. lineatus* clones, the most common *A. matrilineatus* haplotype differs from the most common *A. ludens/lineatus* haplotype by only a single base (K2P distance 0.15%), and the average difference among all known haplotypes of the two groups is 0.5%. Among the known *A. lineatus* clones are eight alleles over six allozyme loci that are not present in *A. matrilineatus* at the type locality; however, in every case, the *A. lineatus* clone is heterozygous for an allele that is found in the *A. matrilineatus* population. These two lines of evidence indicate that while the maternal source of known *A. lineatus* clones was probably not the particular *A. matrilineatus* population present at its type locality, it would nonetheless be considered *A. matrilineatus*. Thus, all the evidence suggests that *A. lineatus* clones arose individually via fertilization of unreduced *A. matrilineatus* eggs by sperm from *A. matrilineatus* males, resulting in triploid zygotes. The relatively high level of heterozygosity in many *A. lineatus* clones (Table S2) and the presence of nuclear alleles not found in *A. matrilineatus* at the type locality suggest the males may have been from another population, or perhaps a very closely related species (either an unknown extant species or one now extinct). The unreduced egg may have been that of a diploid apomictic parthenogen, but demographic evidence makes it seem more likely it was an unreduced egg from a bisexual female, and the transition to apomictic (or at least functionally mitotic) parthenogenesis was coincident with its fertilization (see Section 4.2). *Ameletus ludens* clones are also likely the result of fertilization of unreduced *A. matrilineatus* eggs, but in this case, the sperm came from *A. patriludens* males.

Given our current understanding, do the two obligately parthenogenic lineages *A. ludens* and *A. lineatus*, as well as the bisexuals *A. matrilineatus* and *A. patriludens*, all deserve full species status as presented in the taxonomic section of the present study? While there seems to be no general consensus regarding the taxonomic and nomenclatural treatment of polyploid parthenogenetic lineages of hybrid origin, several authors have proposed reasonable guidelines. Hotz et al. [31] suggest hybrid clonals should be taxonomically recognized when they are distinguished by the combination of parental genomes they contain. On this basis, *A. ludens* and *A. lineatus* qualify as distinct from each other. One of the recommendations proposed by Cole [32] suggests: “When reasonable data indicate that a unisexual taxon reproduces by parthenogenetic cloning and it had a hybrid origin, the taxon should be considered specifically distinct from its parental species. It constitutes a genetically and historically unique, self-perpetuating, separately-evolving entity that is reproductively isolated from its ancestors (although polyploid backcross hybrids might be produced).” Thus, the triploid clonal *A. ludens*, which appears to have arisen via hybridization between an *A. matrilineatus* mother and *A. patriludens* father, should be considered specifically distinct from both parental species. Article 23.8 of the ICZN [33] regarding the application to species-group names established on hybrids states: “A species-group name established for an animal later found to be a hybrid [Article 17] must not be used as the valid name for either of the parental species, even if it is older than all other available names for them.” Thus, the name *A. ludens* is reserved for the hybrid parthenogens.

Although *A. lineatus* may not have been the result of interspecific hybridization per se (i.e., the paternal ancestor would likely be considered a member of the same species as the maternal one), all known *A. lineatus* parthenogens are triploid, and there is no indication they are capable of reverting to bisexuality. Indeed, triploid animals cannot undergo normal meiosis (Stenberg and Saura [34]; although see Nokkala et al. [35]). Thus, *A. lineatus* and *A. matrilineatus* are on distinct and separate evolutionary paths and should be considered separate species.

Are the two bisexuals *A. matrilineatus* and *A. patriludens* sufficiently distinct to be considered species? If the maximum likelihood tree constructed from COI data shown in Figure 19 is correct, *A. janetae* and *A. matrilineatus* are sisters, and thus lumping *A. matrilineatus* and *A. patriludens* would result in paraphyly. However, the bootstrap value for the *A. matrilineatus* plus *A. janetae* node is low. And both the allozyme tree (Figure 19) and morphological evidence (see Section 3.4) suggest *A. matrilineatus* and *A. patriludens* are sisters and *A. janetae* is the outgroup. The 6.12% K2P distance between *A. matrilineatus* and *A. patriludens* is in the range that would be considered separate species by barcode proponents (Hebert et al. [36]) and is similar to a value used recently to help justify the recognition of *Ameletus daliensis* Tong (Li et al. [37]). However, it is less than what was measured between populations of *A. tarteri* that I consider to be conspecific (see Section 4.1.4 below). (A similarly large difference in COI exists among populations which I consider conspecific within the *A. tertius* and *A. browni* groups in the southern Appalachians; Section 4.1.1 and Section 4.1.2.) Although the COI data are equivocal, the fixed allelic difference at allozyme locus *Aat 1* (plus several other nearly fixed differences; see Table S3), combined with the morphological differences documented in Section 3.4, supports the recognition of *A. matrilineatus* and *A. patriludens* as distinct species.

*Ameletus matrilineatus* and *A. patriludens* are clearly very closely related, and if *A. ludens* did in fact arise through hybridization between them, they must have some ability to interbreed. Extant populations are allopatric—the closest known populations of *A. matrilineatus* and *A. patriludens* are separated by 550 km—but in order for those hybridization events that produced *A. ludens* to have occurred, their distributions must have come in contact at some point, probably during the Pleistocene (see Section 4.3). At the type localities of both *A matrilineatus* and *A. patriludens*, the otherwise nearly ubiquitous parthenogens *A. ludens* and *A. lineatus* are conspicuously absent, suggesting the possibility that those parthenogens might have replaced their bisexual progenitors elsewhere. Although other, as yet undetected, populations of *A. matrilineatus* and *A. patriludens* may exist, I can say with confidence that both of these bisexual species are now rare. Yet, they have relatively high expected heterozygosities (H_exp_; 0.16 and 0.13, respectively) compared to other bisexual members of the *A. ludens* group (see Table S2). One plausible explanation might be that the *A. matrilineatus* and *A. patriludens* populations sampled are relicts from a recent contraction and/or fragmentation of formerly large, panmictic populations (perhaps due to displacement by the parthenogens), and that this occurred recently enough that genetic drift has not yet reduced heterozygosity to levels one might expect in a rare endemic.

The fifth and remaining member of the *A. ludens* complex, *A. janetae*, is known only from a small area in eastern West Virginia. Both COI and allozymes indicate very low genetic diversity in this species: H_exp_ = 0.02, and only a single COI haplotype was detected (*n* = 11 individuals). Placement of this species in the *A. ludens* complex is based on morphology (dilation of the genital forceps in the adult male as well as the presence of ventral stripes and speckling of the caudal filaments of the larva) and genetic data (Figure 19). However, both adult males and larvae are readily distinguished from other members of the *A. ludens* complex by characters given in the keys. *Ameletus janetae* does not appear to have been involved in the production of the parthenogenetic *A. ludens* or *A. lineatus*.

#### 4.1.4. The *A. ludens* Species Group: *tarteri* Complex

Members of the *A. ludens* group other than those in the *ludens* complex (Section 4.1.3 above) are discussed here as the *tarteri* complex. Included are three bisexual species, *A. tarteri*, *A. burkei*, and *A. ohioensis*, each with a relatively limited and allopatric distribution, and the widespread triploid parthenogen *A. immaculatus*, which overlaps geographically with all three of the bisexuals. Relationships among these species are uncertain, and this grouping could be paraphyletic, but they are clearly closely related, and at least two of the bisexuals appear to have been involved in the origin of some of the parthenogenetic *A. immaculatus* clones. Adult males of the three bisexual species are distinguishable by differences in genitalia, but females and larvae cannot be identified with certainty on the basis of morphology.

*Ameletus tarteri* is known from scattered localities in eastern West Virginia and adjacent Maryland. Sixty-seven individuals have been sequenced for COI, and these form two very distinct clusters (Figure 19) which differ by up to 10.5% (K2P distance). A group of 52 individuals from northeastern West Virginia and western Maryland forms one cluster labeled “*tarteri* Shay” in that figure. The other cluster, labeled “*tarteri* Ham”, includes 11 from the type locality 100–175 km south in east-central West Virginia, plus five from one of the sites in northeastern West Virginia where “*tarteri* Shay” was also found.

Such a large difference in COI is suggestive of two species. However, members of the two haplotype groups appear identical morphologically. Also, *Ameletus tarteri* is the only *Ameletus* species found in streams with very low pH (~4.5 or lower) and is the only member of the *A. ludens* group that does not have a univoltine life cycle with summer egg diapause, and these features are true for both haplotype groups. But perhaps most importantly, at one site in northeastern West Virginia where both haplotype groups are present, allozyme frequencies show no difference between them, which suggests a single interbreeding population. Thus, I conservatively consider them all *A. tarteri*. Similarly large differences in COI have been documented elsewhere among individuals or populations of conspecifics. For example, Keith and Hedin [38] documented large COI divergence among conspecific spiders in the southern Appalachians, and Dyer et al. [39] found highly divergent COI haplotypes associated with endosymbiont infection within an interbreeding population of *Drosophila quinaria*. Perhaps the simplest explanation in the case of *A. tarteri* is that there were two populations isolated long enough for COI to drift considerably (but not long enough to result in reproductive incompatibility), followed by a recent reconnection and introgression of the “*tarteri* Ham” haplotype into a “*tarteri* Shay” population.

*Ameletus ohioenesis* is known from southeastern Ohio and a few sites in adjacent West Virginia and has a known range similar in size to that of *A. tarteri*. Eight *A. ohioenesis* haplotypes were found at the type locality and a ninth from a site 30 km away in West Virginia. K2P distances among haplotypes averaged 0.6% (range 0.15–1.25%). The species coexists with *A. lineatus* and *A. immaculatus*, but little else is known of its biology. *Ameletus burkei* is presently known only from the type locality in southwest Virginia.

Similarly to the situation seen in the *A. ludens* complex, the clonal parthenogenetic member of the *A. tarteri* complex, *A. immaculatus*, is by far the most common and widely distributed. *Ameletus immaculatus* ranges from northern New England south to North Carolina and west to at least western West Virginia and southern Ohio, whereas the known bisexuals all have very limited distributions (see above). Compared to *A. ludens* and *A. lineatus*, the bisexual ancestry of *A. immaculatus* is less clear.

COI in *A. immaculatus* shows little variation and thus a single maternal ancestor species is implicated. Of the 121 *A. immaculatus* for which we have COI sequences, the majority (*n* = 65) share a single haplotype, and these individuals cover the entire known geographic range of the species. Another 21 have the second most common sequence, differing from the common haplotype by a single base. Twenty-six others have one of four other sequences, again each differing from the common haplotype by a single base. Seven singletons have two substitutions, one has six, and one has eight substitutions. Ignoring the two outliers, the mean K2P distance among all the other haplotypes is 0.22% (the two outliers differed from the common haplotypes by an average of 1.2% and 1.6%, and from each other by 2.1%).

In contrast to the near-uniformity of mitochondrial COI, nuclear gene diversity in *A. immaculatus* is high, with 36 known allozyme MLGs. For example, Figure 17F illustrates variation at the *Gpi* allozyme locus for individuals of *A. immaculatus* and *A. tarteri* collected from a single site where they co-occur. In this case, five *Gpi* alleles were found among the four *A. immaculatus* clones present. Eight *Gpi* alleles have been found in the species overall, all of which occur in one or more of the *A. tarteri* complex sexuals. Heterozygosity in *A. immaculatus* is also high, averaging 0.247 (range 0.136–0.455; Table S2). The mean DNA content per nucleus was determined by flow cytometry for *A. immaculatus* to be 1.10 pg (*n* = 37), which is almost exactly 50% greater than that determined for its closest maternal relative, *A. burkei* (0.74 pg; *n* = 3m, 18f), indicating triploidy in *A. immaculatus*. Together, these measures make a convincing case for a hybrid origin for most or all clones.

The maternal ancestor of *A. immaculatus* is presently unknown, but among the known members of the *A. tarteri* complex, the most similar COI haplotypes are found in *A. burkei* (K2P distance ~3%). The two coexist at the type locality of *A. burkei* in southwest Virginia, where a total of 15 *A. immaculatus* and 51 *A. burkei* have been sequenced. At that site, only two *A. immaculatus* haplotypes were detected, with 14 of the 15 individuals having the haplotype that is most common for the species overall and found throughout its range. For the *A. burkei*, six haplotypes were found. Two that differ by only a single base accounted for 45 of those 51 (27 individuals of one type and 18 of the other). The four other haplotypes each differed from the common one by a single base. K2P distances among *A. burkei* haplotypes ranged from 0.15% to 0.48%. Pairwise comparisons between *A. immaculatus* and *A. burkei* haplotypes from that site revealed 14 to 20 substitutions (K2P distances of 2.1–3.3%).

Although COI sequences among *A. immaculatus* clones indicate a single (but presently unknown) maternal ancestor species, in the NJ tree (shown in Figure 19), these clones (allozyme MLGs) are scattered amongst the three *A. tarteri* complex bisexual species, which suggests that not all clones share the same paternal species. In some cases, clones are most similar to the particular bisexual species with which they were found. For example, all 11 *A. immaculatus* clones found together with *A. burkei* at its type locality at Burke’s Garden, Virginia, cluster with that species in the tree. This is primarily due to similarities at two allozyme loci, *Mpi* and *aGpdh* where alleles present in most other *A. immaculatus* clones are missing in these Burke’s Garden clones, being replaced by alleles that are found in *A. burkei* but not in one or both of the other *A. tarteri* complex bisexuals. Similarly, one *A. immaculatus* clone found along with *A. ohioensis* at the latter’s type locality clusters very closely with that species (see Figure 19). This clone is heterozygous for alleles only found in *A. ohioensis* at loci *Mdh1*, *Aat2*, *Gpi*, *Mpi*, and *Pro*. Except for the *Gpi* allele, these are missing from all other known *A. immaculatus* clones.

In the above cases it seems *A. immaculatus* clones were the result of hybridization events between a member of a single maternal ancestral species (which is unknown and possibly extinct) and a male *A. burkei* or *A. ohioenensis*, but for the majority of *A. immaculatus* clones depicted in Figure 19 there is no clear indication of a particular paternal source. While there are large differences in allele frequency among the five *A. ludens* group bisexuals for some allozyme loci (Table S3), there are few private alleles, especially ones at high frequency or fixation, which means there is a scarcity of markers that would allow us to definitively identify parental sources for all the parthenogens.

With the exception of streams having very low pH, in which *A. tarteri* is the only *Ameletus* species present, at sites where I have found any of the three bisexual members of the *A. tarteri* complex (*A. tarteri*, *A. burkei*, or *A. ohioensis*), *A. immaculatus* is also found. But even though at least two of these bisexuals were likely involved paternally in the origins of *A. immaculatus* clones, there is no evidence of ongoing hybridization. If a male of one of these bisexuals were to mate with and fertilize eggs of a triploid *A. immaculatus* parthenogen, those offspring should be tetraploid. It seems the most likely places for such backcrosses to have occurred would be those sites mentioned above, where *A. burkei* or *A. ohioensis* coexist with *A. immaculatus* clones, but all the clones tested from those sites have been triploid.

Hotz et al. [31] recommend against lumping different parental species combinations or different allopolyploidy levels into single names. All known *A. immaculatus* clones are triploid, so combining different ploidy levels is not an issue, but what I here consider *A. immaculatus* likely includes clonal lineages with different paternal sources. However, in most cases, the paternal species cannot be determined with certainty. Members of this inclusive concept of *A. immaculatus* are morphologically uniform, and their occurrence is predictable (only found in the smallest headwater streams). Thus, in the absence of definitive knowledge of their bisexual heritage, it seems for now that a conservative taxonomic approach is warranted.

### 4.2. Parthenogenesis and Its Evolution in Ameletus

Though rare in animals generally, parthenogenesis is common in Ephemeroptera, where nearly half of the 136 species that have been tested are able to reproduce parthenogenetically [40]. For the large majority of these, parthenogenesis appears to be facultative. For example, in seven species of bisexual baetids, all of which had essentially 1:1 sex ratios in the field, Funk et al. [41] documented mean hatch rates for unfertilized eggs ranging from 40 to 84%. The parthenogenetic mechanism in these species was automictic (most likely central fusion with crossing over) which resulted in a heterozygosity loss of 10–24% per parthenogenetic generation, and those authors hypothesized that a reduction in fitness resulting from heterozygosity loss explained, at least in part, why these species remained predominantly bisexual.

A much smaller number of mayflies are obligate thelytokous parthenogens, but this condition is still more common among mayflies than for most other insect groups [40]. For example, in White Clay Creek, Pennsylvania, USA, 8 (16%) of the 50 known mayfly species are unisexual. Three of these represent species that are entirely parthenogenetic (*Neocloeon triangulifer*, *Ameletus ludens*, and *A. lineatus*), and five are unisexual populations of species for which bisexual populations are known to exist elsewhere (*Acerpenna macdunnoughi*, *Anafroptilum minor*, *Diphetor hageni, Eurylophella funeralis*, and *Ephemera varia*). All of these obligate parthenogens have been shown to reproduce clonally [41]. And except for the *Ameletus*, allozyme data suggest they are all diploid. New data on genome size (mean pg DNA/nucleus) derived from flow cytometry now confirms diploidy for three of these: *N. triangulifer* (unisexual 0.32 ± 0.01, *n* = 6; bisexual [sister species *N. alamance*] 0.39 ± 0, *n* = 2); *A. macdunnoughi* (unisexual 0.40 ± 0, *n* = 2; bisexual 0.42, *n* = 1); and *E. funeralis* (unisexual 0.64 ± 0.01, *n* = 3; bisexual 0.61 ± 0.03, *n* = 7).

Although polyploid parthenogens, which are generally of hybrid origin, are found in other insect groups such as weevils and stick insects, as well as in vertebrates [42], the three parthenogenetic *Ameletus* documented herein represent the first such cases to be discovered among the Ephemeroptera. All parthenogenetic *Ameletus* tested have been triploid, and high levels of heterozygosity indicate a hybrid origin, at least for *A. ludens* and *A. immaculatus*. A number of hypothetical pathways to allopolyploidy have been proposed (see Choleva and Janko [43]). One possibility is that the first stage was an automictic diploid parthenogen, which then evolved to diploid apomixis, and finally the fertilization of an apomictic diploid egg by a haploid sperm (from a related species) produced the triploid apomictic parthenogens we find today. In the case of *Ameletus*, this pathway seems unlikely for two reasons. As pointed out by Saura et al. [44], many modes of automixis enforce homozygosity, likely an unpromising start for parthenogenetic reproduction (see Funk et al. [41]; although there exist numerous examples of diploid mayfly parthenogens that have maintained a level of heterozygosity comparable to their bisexual ancestors—see paragraph above). Secondly, we might expect to find both diploid apomictic forms as well as higher-level polyploids (tetraploid or higher) from subsequent backcrosses among these *Ameletus*. But only triploids have been found. From the patterns we see today, it seems more likely that hybridization either briefly preceded or was coincident with the transition to polyploid parthenogenesis. For example, an initial hybridization event may have led to the production of an unreduced female gamete (unreduced gametes are common in species crosses or crosses involving different populations of a species; Saura et al. [44]) which was then fertilized by a haploid gamete from one of the parental species or even a third species (the “Genome Addition Hypothesis” of Choleva and Janko [43]). Another possibility is that an unreduced gamete resulting from defective meiosis in a non-hybrid mother was fertilized by a haploid sperm from a related species, resulting in an instant transition to apomictic polyploid parthenogenesis (the “Spontaneous Allopolyploid Origin” of Choleva and Janko [43]).

Each of the *Ameletus* parthenogens have very low COI diversity, with the majority of clones sharing a single haplotype, indicating a recent, common maternal origin. However, each also consists of many different clones (allozyme MLGs), and this, combined with the fact that most alleles found in these MLGs are present in at least one of the bisexuals (and thus presumably did not arise following the transition to asexuality), suggests the various clones may have arisen independently. *Warramaba virgo* is a well-studied diploid parthenogenetic grasshopper, widespread in southern Australia, which arose from hybridization of the bisexuals *W. whitei* and *W. flavolineata*. From data that included COI and allozyme patterns similar to what we see in *Ameletus*, investigators had concluded that much of the clonal diversity in *W. virgo* could be explained by repetitive hybrid origins (Honeycutt and Wilkinson [45]; Kearney and Blackett [46]). However, a more recent study based on single-nucleotide polymorphisms (SNPs) led Kearney et al. [47] to conclude that all *W. virgo* were actually the result of a single hybrid mating that occurred at least 0.25 million years ago, and that the observed genetic variability is post-formational. In the case of *Ameletus*, there had to have been at least two events involving females of the bisexual *A. matrilineatus*, one of which gave rise to the parthenogen *A. lineatus* and another that gave rise to *A. ludens*. In the case of *A. immaculatus*, there must have been at least three founding events (see Section 4.1.4).

If the *Ameletus* parthenogens arose by way of something like the Spontaneous Allopolyploid Origin model, once the parental species came into contact the limiting factor for these events to occur may have been the production of those unreduced eggs that are able to make the transition to functional apomixis when fertilized by a heterospecific. If the females possessing this propensity were restricted to a single population, this might be reflected in the present-day dominance of a single COI haplotype in each of the parthenogens. Perhaps once the prospects met up during Pleistocene range shifts, many of these events occurred, and most of the allozyme variants came from the males. And most or all of the COI variants we see now in the parthenogens (other than the dominant one), the bulk of which differ from the dominant haplotype by only a single base, may have arisen subsequently.

### 4.3. Geographic Parthenogenesis the A. ludens group

The relative uniformity of COI haplotypes in *Ameletus* parthenogens suggests they arose recently, likely during the Pleistocene, when range shifts propelled by glacial advances brought formerly distant species/populations together, which then hybridized to form the clones we see now. These parthenogens have been extremely successful, as indicated by their near ubiquity over a broad geographic area compared with their bisexual relatives/progenitors, all of which have quite limited distributions. Such a pattern, termed geographical parthenogenesis, appears in many parthenogenetic/bisexual complexes and is often associated with polyploidy, hybridization or both (Kearney [48]). One obvious advantage parthenogens have over their bisexual relatives might be their ability to more rapidly recolonize following glacial retreat. Theoretically, asexuals have double the reproductive rate of their bisexual relatives (every individual produces eggs), and a single female can establish a new population without having to depend on the availability of males. However, *Ameletus* parthenogens are also polyploid and of hybrid origin, so their success might not be explained simply by the demographic advantages of asexuality. In a study of weevils that included diploid bisexuals and parthenogens of various ploidy levels (diploid, triploid, and tetraploid), Stenberg et al. [49] concluded that it was an increase in ploidy level and not the benefits of asexual reproduction that conferred to polyploid parthenogens the advantage over their diploid sexual relatives. Kearney [48] argued that hybridization might play a primary role in the success of parthenogens by creating genetic diversity which, when stabilized by parthenogenesis, could provide important colonizing advantages.

Although it is difficult to tease apart the relative importance of hybridization, polyploidy, and parthenogenesis itself to the demographic success of *Ameletus* parthenogens compared with their bisexual progenitors, the present-day rarity of the latter is likely not simply an artifact of their lower dispersal abilities. The conspicuous absence of the parthenogens *A. ludens* and *A. lineatus* from sites where their sexual progenitors *A. matrilineatus* and *A. patriludens* are found suggests the possibility that the parthenogens have actually displaced the bisexuals elsewhere. While we do find *A. immaculatus* coexisting with bisexuals that may have been involved in its paternal ancestry, its maternal ancestor has not yet been found, and may be extinct. Vrijenhoek’s Frozen Niche Variation model (summarized in Vrijenhoek and Parker [50]) provides a theoretical pathway for such an outcome, whereby a sexual population exhibits a range of genetic variation in its ability to utilize a natural resource, and this variation is effectively carved up and frozen among clones. Natural selection eliminates clones with too much overlap with each other or the centrally distributed sexual ancestor, allowing coexistence if the rate of clonal origin is low. However, when the rate of clonal formation is too high, the clones might eclipse resource use by the sexual ancestor, leading to its extinction. Another possibility is that parthenogenetic female *Ameletus* compete for the attention of bisexual males such that eggs of many bisexual females go unfertilized. A technique I have used to induce oviposition in other parthenogenetic mayflies, i.e., dropping the imago onto the surface of water in a small glass vessel, almost never results in oviposition by *Ameletus* parthenogens. However, in the field, I have captured *A. ludens* and *A. lineatus* females in a descending flight from a height of at least 3 m above a stream, and when these females were dropped into such vessels, they did oviposit. This flight may be a relictual swarming behavior, and if males were present, a mating could result. If parthenogens became numerous enough locally, a decline in the bisexuals might result from those females failing to procure a mate.

The success of *Ameletus* parthenogens in the eastern Nearctic seems to extend beyond just the recolonization of deglaciated territory and possible displacement of their bisexual relatives. *Ameletus* generally are cold stenotherms whose larvae are not found in waters warmer than about 15 °C. However, the eastern Nearctic parthenogens can be found in relatively warm, lowland stream habitats whose summer temperature rises well above 15 °C and that probably would not have supported the ancestral *Ameletus*. Most members of the *A. ludens* group (which includes the bisexual progenitors of the three parthenogens) have an obligate summer egg diapause, which enables larvae to avoid exposure to warm summer conditions. Genetic variability among the sexual progenitors may have resulted in the production of clones with varying diapause lengths, some of which are better suited to streams that are warm for longer periods.

### 4.4. Future Work

Several lines of inquiry could lead to a better understanding of the *A. ludens* group. Discovery of a population of the maternal ancestor of *A. immaculatus* (which should be identifiable by COI) and utilization of more modern nuclear genetic techniques to accurately identify its paternal ancestor(s) could enable the testing of hypotheses concerning the hybrid origin of parthenogenetic clones. Copulation in *Ameletus* is easily induced in the laboratory (Figure 4G), and hybrid matings might produce illuminating results.

Some *Ameletus*, especially parthenogenetic members of the *A. ludens* group, are frequently found in streams that become dry part of the year. These have even been listed as indicators of intermittent flow (Fritz et al. [51]). Because stream channel drying is most likely to occur during summer and early autumn when these *Ameletus* are typically in egg diapause, one might assume diapausing eggs are able to resist or tolerate desiccation. However, in tests I have performed, where diapausing eggs were dried slowly over a two-week period and subsequently re-wetted, none have survived (in parallel tests with *Siphlonurus marshalli*, which also inhabits streams with intermittent flow, eggs survived this treatment). Eggs of many mayflies (including *S. marshalli*) are ovoid and have various structures that attach them firmly to substrates (Brittain [52]), but eggs of some *Ameletus*, including all members of the *A. ludens* group, are flattened, in the form of an ellipse-like biconvex lens (Zloty and Pritchard [10]; Kluge [11]) and are devoid of conspicuous attachment devices (pers. obs.). It may be these eggs somehow work their way down into the hyporheic, where they are able to remain wet during periods when the surface is dry.

In preliminary studies (Sweeney et al. [53] and unpublished), the duration of egg diapause in *A. ludens* group parthenogens appeared to be independent of incubation temperature, and to vary predictably over a latitudinal gradient, with that of clones from southern regions lasting significantly longer (presumably to accommodate longer summers). And because for a given clone, the length of diapause was consistent over two generations in the laboratory, its duration might be genetically fixed. However, more recent tests indicate that a delay in seasonal temperature drop can delay the termination of diapause and result in high rates of mortality. For clones maintained in the laboratory, diapause length appears to change after several generations, perhaps in response to thermal conditions. These studies are ongoing and will be reported on in the future.

## Data Availability

DNA sequences are available on BOLD or upon request from the author.

## Supplementary Materials

The following supporting information can be downloaded at https://www.mdpi.com/article/10.3390/insects16050530/s1, Table S1: Enzyme systems; Table S2: Heterozygosity values; Table S3: Allele frequencies; Table S4: Specimen list; Table S5: fasta file with the most common COI sequence occurring in *Ameletus immaculatus*; Table S6: fasta file with the most common COI sequence occurring in *Ameletus ludens* and *A. lineatus*. References [54,55,56,57,58,59] are cited in the Supplementary Materials.
