# Supplementary material for: Ameletus Mayflies (Ephemeroptera: Ameletidae) of the Eastern Nearctic
Source: Insects. 2025 May 16;16(5):530. doi: 10.3390/insects16050530 (PMC12111989; doi:10.3390/insects16050530)
Supplement: Supplementary file 1 [file insects-16-00530-s001.zip › Table S1.pdf]

Table S1. Enzyme systems used for *Ameletus* allozyme work. E.C. = Enzyme Commision.

E.C. no.	locus	acronym	#loci detectable	#loci scorable	buffer system[a]	reference for stain recipe[c]
1.1.1.8	a-glycerophosphate dehydrogenase	*a-Gpdh*	1	1	P-7	1
1.1.1.27	Lactate dehydrogenase	*Ldh*	1	0	P-7	1
1.1.1.37	Malate dehydrogenase	*Mdh*	2	2	TC-6/P-7	1
1.1.1.40	Malic enzyme	*Me*	1	1	P-7	1
1.1.1.42	Isocitrate dehydrogenase	*Isdh*	2	2	P-7	1
1.1.1.44	Phosphogluconate dehydrogenase	*6pgd*	1	1	P-7	1
1.1.1.47	Glucose dehydrogenase	*Gdh*	1	0	P-7	1
1.1.1.49	Glucose-6-phosphate dehydrogenase	*G6pdh*	1	1	TC-6	1
1.1.1.204	Xanthene dehydrogenase	*Xdh*	1	0	P-7	4
1.2.1.12	Glyceraldehyde-phosphate dehydrogenase	*G3pdh*	1	1	P-7	1
1.2.3.1	Aldehyde Oxidase	*Ao*	1	0	P-7	3
1.15.1.1	Superoxide dimutase	*Sod*	2	2	TEB9/8	1
2.6.1.1	Aspartate aminotransferase	*Aat*	2	2	P-7	3
2.7.1.1	Hexokinase	*Hex*	1	1	P-7	1
2.7.1.40	Pyruvate kinase	*Pk*	1	1	TEB9/8	5
2.7.3.3	Arginine kinase	*Ark*	1	1	P-7	6
2.7.4.3	Adenylate kinase	*Adk*	1	1	P-7	1
3.1.1.1	Esterases	*Est*	5	2	TEB-8	1
3.1.3.2	Acid phosphatase	*Acp*	1	0	TC-6	1
3.4.11.4	Tripeptide aminopeptidase	*Tri*	2	1	TEB-8	1[b]
3.4.13.9	Proline dipeptidase	*Pro*	1	1	TEB-8	1[b]
3.4.13.11	Dipeptidase	*Dip*	2	2	TEB-8	1[b]
3.5.4.3	Guanine deaminase	*Gda*	1	1	P-7	1
3.5.4.4	Adenosine deaminase	*Ada*	1	1	TEB9/8	1
4.1.2.13	Aldolase	*Ald*	1	1	P-7	1
4.2.1.3	Aconitase	*Acon*	2	2	P-7	1
5.3.1.1	Triose phosphate isomerase	*Tpi*	1	1	P-7	1
5.3.1.8	Mannose phosphate isomerase	*Mpi*	1	1	P-7	1
5.3.1.9	Glucose phosphate isomerase	*Gpi*	1	1	TEB-8	1
5.4.2.2	Phosphoglucomutase	*Pgm*	1	1	P-7	1

[a] buffer systems are as follows:

P-7; electrode = 0.067M sodium phosphate (monobasic), 0.133M sodium phosphate (dibasic), pH 7.0; gel = electrode buffer 1:20
TC-6; electrode = 0.24M tris, 0.085M citric acid, pH 6.0; gel = electrode buffer 1:28
TEB9/8; electrode = 0.5M tris, 0.65M boric acid, 0.018M EDTA, pH 8.0; gel = 0.087M tris, 0.014M boric acid, 0.001M EDTA, pH 9.0
TEB-8; electrode = 0.5M tris, 0.65M boric acid, 0.018M EDTA, pH 8.0; gel = 0.05M tris, 0.1M boric acid, 0.002M EDTA, pH 8.0

Table S1. Enzyme systems used for *Ameletus* allozyme work. E.C. = Enzyme Commision.

[b] substrates for *Tri*, *Pro*, and *Dip* were leucylglycylglycine, phenylalanylproline, and leucylalanine respectively
[c] 1= Harris & Hopkinson (1976), 3= Richardson, et al (1986), 4= Shaw & Prasad (1970), 5= Brewer (1970), 6= Zurwerra et al (1986)

Brewer, G. J. 1970. *An introduction to isozyme techniques* . Academic Press, New York.

Harris, H. and D. A. Hopkinson. 1976. *Handbook of enzyme electrophoresis in human genetics* . American Elsevier, New York.

Richardson, B. J., P. R. Baverstock and M. Adams. 1986. *Allozyme electrophoresis: A handbook for animal systematics and population studies* . Academic Press, Sidney.

Shaw, C. R. and R. Prasad. 1970. Starch gel electrophoresis of enzymes - a compilation of recipes. *Biochem. Genet.* **4:** 297-320.

Zurwerra, A., I. Tomka and G. Lampel. 1986. Morphological and enzyme electrophoretic studies on the relationships of the European *Epeorus* species (Ephemeroptera, Heptageniidae). *Syst. Entomol.* **11:** 255-266.
