# Supplementary material for: Ameletus Mayflies (Ephemeroptera: Ameletidae) of the Eastern Nearctic
Source: Insects. 2025 May 16;16(5):530. doi: 10.3390/insects16050530 (PMC12111989; doi:10.3390/insects16050530)
Supplement: Supplementary file 1 [file insects-16-00530-s001.zip › Table S2.pdf]

Table S2. Heterozygosity values (allozyme data, 22–28 loci) for eastern Nearctic *Ameletus* species (other than *A. subnotatus* group). Heterozygosity figures for parthenogens (*A. ludens, A. lineatus, A. immaculatus*) are direct count (means) and *n* equals number of clones. For bisexual species (all others) heterozygosity values are H_{exp} (Nei 1978) and *n* equals the number of individuals sampled from that population.

locality	species group	species-population	Hetero-zygosity	*n*	min	max
various	*ludens*	*ludens*	0.273	18	0.182	0.364
various	*ludens*	*lineatus*	0.185	14	0.091	0.273
various	*ludens*	*immaculatus*	0.247	36	0.136	0.455
Virginia (type)	*ludens*	*matrilineatus*	0.161	37		
New York (type)	*ludens*	*patriludens*	0.126	25		
West Virginia (type)	*ludens*	*janetae*	0.018	7		
Virginia (type)	*ludens*	*burkei*	0.089	55		
West Virginia (type)	*ludens*	*tarteri*-Ham	0.065	60		
West Virginia	*ludens*	*tarteri*-Shay	0.093	29		
Ohio (type)	*ludens*	*ohioensis*	0.072	62		
Maine	*browni*	*browni*-ME	0.015	10		
Vermont	*browni*	*browni*-VT	0.036	34		
Pennsylvania	*browni*	*browni*-PA	0.036	18		
Vermont	*browni*	*rebucki*-VT	0.114	54		
Pennsylvania (type)	*browni*	*rebucki*-PA	0.106	53		
West Virginia	*browni*	*rebucki*-WV	0.137	15		
Virginia	*browni*	*cryptostimulus*-Big	0.040	29		
Virgina (type)	*browni*	*cryptostimulus*-LSC	0.070	45		
South Carolina	*browni*	*cryptostimulus*-SC	0.075	19		
Quebec	*tertius*	*tertius*-Pig	0.021	4		
Quebec	*tertius*	*tertius*-Mont	0.056	2		
New York	*tertius*	*tertius*-Hun	0.044	5		

Nei M. 1978. Estimation of average heterozygosity and genetic distance from a small number of individuals. Genetics 89:583-590.
